# Photochemical generation of the 2,2,6,6-tetramethylpiperidine-1-oxyl (TEMPO) radical from caged nitroxides by near-infrared two-photon irradiation and its cytocidal effect on lung cancer cells

**DOI:** 10.3762/bjoc.15.84

**Published:** 2019-04-10

**Authors:** Ayato Yamada, Manabu Abe, Yoshinobu Nishimura, Shoji Ishizaka, Masashi Namba, Taku Nakashima, Kiyofumi Shimoji, Noboru Hattori

**Affiliations:** 1 Department of Chemistry, Graduate School of Science, Hiroshima University, 1-3-1 Kagamiyama, Higashi-Hiroshima, Hiroshima 739-8526, Japan; 2 Hiroshima Research Centre for Photo-Drug-Delivery Systems (HiU-P-DDS), Hiroshima University, 1-3-1 Kagamiyama, Higashi-Hiroshima, Hiroshima 739-8526, Japan; 3 JST-CREST, K’s Gobancho 6F, 7, Gobancho, Chiyoda-ku, Tokyo 102-0075, Japan; 4 Graduate School of Pure and Applied Sciences, University of Tsukuba, 1-1-1 Tennoudai, Tsukuba, Ibaraki 305-8571, Japan; 5 Department of Molecular and Internal Medicine, Graduate School of Biomedical & Health Sciences, Hiroshima University, 1-2-3 Kasumi, Minami-ku, Hiroshima, Hiroshima 734-8551, Japan

**Keywords:** caged compound, nitroxide, photolysis, radical, theranostics, two-photon

## Abstract

Novel caged nitroxides (nitroxide donors) with near-infrared two-photon (TP) responsive character, 2,2,6,6-tetramethyl-1-(1-(2-(4-nitrophenyl)benzofuran-6-yl)ethoxy)piperidine (**2a**) and its regioisomer **2b**, were designed and synthesized. The one-photon (OP) (365 ± 10 nm) and TP (710–760 nm) triggered release (i.e., uncaging) of the 2,2,6,6-tetramethylpiperidine-1-oxyl (TEMPO) radical under air atmosphere were discovered. The quantum yields for the release of the TEMPO radical were 2.5% (**2a**) and 0.8% (**2b**) in benzene at ≈1% conversion of **2**, and 13.1% (**2a**) and 12.8% (**2b**) in DMSO at ≈1% conversion of **2**. The TP uncaging efficiencies were determined to be 1.1 GM at 740 nm for **2a** and 0.22 GM at 730 nm for **2b** in benzene. The cytocidal effect of compound **2a** on lung cancer cells under photolysis conditions was also assessed to test the efficacy as anticancer agents. In a medium containing 100 μg mL^−1^ of **2a** exposed to light, the number of living cells decreased significantly compared to the unexposed counterparts (65.8% vs 85.5%).

## Introduction

Nitroxides (aminoxyl radicals) possess a delocalized unpaired electron and exhibit negligible dimerization reactivity, making them persistent open-shell species [1–4]. In addition to their ease of handling, nitroxides are highly sensitive to electron paramagnetic resonance (EPR) spectroscopy and redox reactions. Therefore, nitroxides have been developed and utilized in diverse and crucial applications, not only in chemistry, but also in biology, physiology, and energy sciences. These applications include spin-labels [5–7], fluorophore-nitroxide probes [8], contrast agents in magnetic resonance imaging (MRI) [9], polarization transfer agents for nuclear magnetic resonance (NMR) [10–13], and radical batteries [14–15]. Furthermore, the efficient synthesis of polymers with narrow molecular mass distributions has been accomplished using nitroxides as a mediator, i.e., so-called nitroxide-mediated polymerization (NMP) [16–20], and the nitroxide-mediated synthesis of ketones from alcohols is also well utilized in organic synthesis [21–25]. The huge number of studies concerning nitroxides clearly indicates the importance of new methods of generating nitroxides for the future development of science and technology. Notably, in physiological studies [26–32], spatiotemporal control of nitroxide generation is a key approach for investigating the role of redox-active nitroxides in mediating oxidative stress in organisms [27–32].

**Scheme 1:**
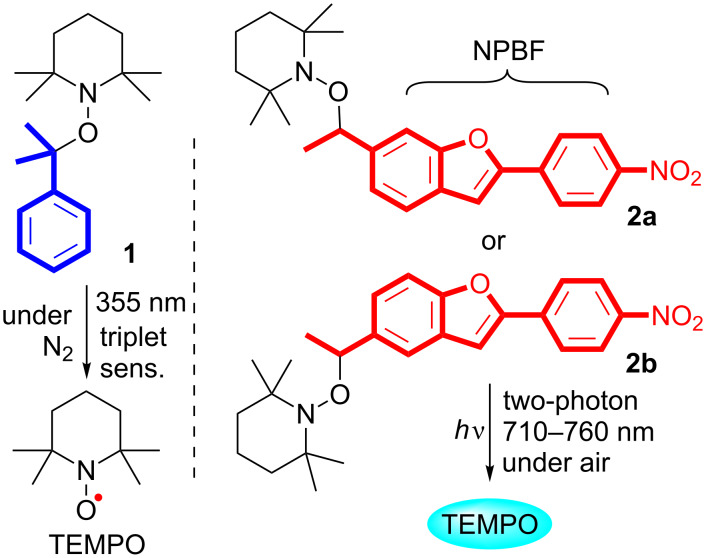
Photochemical generation of TEMPO radical.

In 1997, Scaiano and co-workers reported the triplet-xanthone sensitized generation of the 2,2,6,6-tetramethylpiperidine-1-oxyl (TEMPO) radical from alkoxyamine **1** under ultraviolet (355 nm) irradiation (Scheme 1) [33]. De-aerated conditions are necessary for the triplet-sensitized generation of TEMPO due to the triplet quenching ability of O_2_. The polymerization reactions were initiated via photochemical reaction [34–36]. For physiological studies, however, the photochemical release of nitroxides should be achieved in the presence of O_2_. Thus, the triplet sensitized method may not be useful for physiological studies. The application of alkoxyamines as theranostic agents [37–40] has been proposed and reported by Brémond and co-workers [41–42].

Near-infrared (NIR) photons are excellent light sources in physiological studies as this wavelength of light is less harmful to living tissue than ultraviolet irradiation. Deeper penetration of NIR photons into biological samples is possible using NIR radiation with wavelengths of 650–1050 nm (= 27–44 kcal mol^−1^). However, in general, chromophores do not absorb at such long wavelengths and the photon energy is too low for bond-cleavage reactions to generate (i.e., uncage) functional molecules. For example, the bond-dissociation energy of the weak PhCH_2_–OPh, linkage is reported to be 52.1 kcal mol^−1^ [43]. These issues can be solved by using the NIR-two-photon (TP) excitation technique [44], in which a molecule is electronically excited to the same state generated by one-photon (OP) excitation in the UV–vis region [45]. In addition to the advantages of TP excitation, three-dimensional control of the electronic excitation is possible because the probability of TP excitation is proportional to the square of the light intensity [46]. The light-induced generation of nitroxides using the TP excitation technique, i.e., the concentration jump of nitroxides, is one promising method of exploring the role of these species in life phenomena [47–54] and of promoting site-selective chemical reactions such as polymerization. Very recently, Guillaneuf and co-workers reported the two-photon-induced release of nitroxides in a materials science study [55].

In the last decade, we developed a TP-responsive photo-labile protecting group [56–58] with simple cyclic stilbene structures such as 2-(4-nitrophenyl)benzofuran (NPBF) that absorb in the NIR region of 710–760 nm for the uncaging of bioactive substances such as glutamate and Ca^2+^ [59–64]. Herein, we report the synthesis of new caged nitroxides (nitroxide donors) **2a** and **2b** having the TP-responsive NPBF chromophore and the NIR TP-triggered generation of the 2,2,6,6-tetramethylpiperidine-1-oxyl (TEMPO) radical under atmospheric conditions using these species (Scheme 1). Because free radicals are cytotoxic due to their strong DNA-damaging activity [65], they play important roles as anticancer therapeutic agents [66]. Among the free radicals, nitroxides including the TEMPO radical have unique properties, where they can act not only as radical scavengers, but also as anticancer agents [67]. Due to the unique properties described above, nitroxides are not toxic to normal host cells and exhibit toxicity only to tumor cells. Thus, nitroxides are ideal candidates as anticancer therapeutic agents. Based on this knowledge, the cytocidal effect of the radical released from compound **2a** on lung cancer cells was tested in vitro, in addition to the fundamental study.

## Results and Discussion

**Scheme 2:**
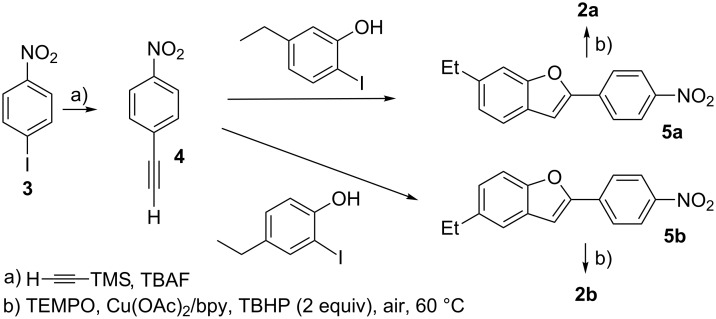
Synthesis of caged nitroxides **2a** and **2b**.

The caged-TEMPOs **2a** and **2b** were synthesized as shown in Scheme 2. The new compounds, 5-ethyl- and 6-ethyl-2-(4-nitrophenyl)benzofuran (**5a** and **5b**), were synthesized from 1-ethynyl-4-nitrobenzene (**4**) that was prepared from the commercially available 1-iodo-4-nitrobenzene (**3**) [68]. The TEMPO moiety was introduced at the benzylic position of **5a** and **5b** using the copper-catalyzed radical reaction in the presence of *tert-*butyl hydroperoxide (TBHP) to afford **2a** and **2b** in 38% and 52% yield, respectively [69]. The caged TEMPOs **2a** and **2b** were thermally stable in benzene below 320 K (47 °C), as confirmed by electron paramagnetic resonance (EPR) spectroscopic analysis. Significant thermal decomposition of **2a** and **2b** was observed at ≈340 K (67 °C), as indicated by the typical EPR signals (see Supporting Information File 1, Figure S1).

The photophysical data for the new compounds **2a**,**b** and **5a**,**b** are summarized in Table 1. The absorption maxima of compounds **2** and **5** were observed at ≈370 nm with a molar extinction coefficient ε ≈20000 M^−1^ cm^−1^ in both benzene and DMSO. The emission profile showed a significant solvent effect. The fluorescence quantum yields in DMSO of **5a** and **5b** were determined to be 16.1 and 8.6%, respectively, although no emission was observed from these compounds in non-polar benzene, indicating that the excited state has zwitterionic character. The charge transfer transition was supported by time-dependent density functional theory (TD-DFT) calculations for **5a** at the CAM-B3LYP/6-31G(d) level of theory (Supporting Information File 1, Figure S2). The fluorescence quantum yields of caged-TEMPO **2a** and **2b** were found to be 2.9 and 2.2% in DMSO, which are much smaller than those of **5a** and **5b**, respectively, suggesting the chemical reactivity of the singlet excited states of **2a** and **2b**.

**Table 1:** Photophysical data for **2a**, **2b**, **5a**, and **5b** in benzene (DMSO).

Entry		λ_abs_ [nm]^a^	ε [M^−1^ cm^−1^]	λ_em_ [nm]^b^	Φ_f_ × 10^2 c^	τ [ps]^d^

1	**2a**	371 (375)	24800 (23100)	– (576)	≈0.0 (2.9)	– (220, 1370)^e^
2	**2b**	366 (370)	23000 (23400)	– (564)	≈0.0 (2.2)	– (390, 890)^f^
3	**5a**	372 (378)	23800 (20000)	– (577)	≈0.0 (16.1)	– (1430)
4	**5b**	367 (372)	22300 (19000)	– (563)	≈0.0 (8.6)	– (870)

^a^Absorption maximum of **2a**, **2b**, **5a**, **5b**. ^b^Emission maximum of **2a** (1.18 × 10^−6^ M), **2b** (1.18 × 10^−6^ M), **5a** (1.16 × 10^−6^ M), **5b** (1.12 × 10^−6^ M). ^c^Fluorescence quantum yields. The standard sample 9,10-diphenylanthracene (Φ_f_ = 0.91) was used for determining the quantum yields. ^d^Fluorescence lifetime monitored at 560 nm. The concentrations were the same as those used for the fluorescence measurements. ^e^Each contribution is 57% and 43%, respectively. ^f^Each contribution is 70% and 30%, respectively.

Time-correlated single photon counting (TCSPC) measurement was performed at 298 K in DMSO to estimate the fluorescence lifetime (τ) of **2** and **5** (Table 1). Single-exponential decay curves were observed for **5a** and **5b**, respectively (Supporting Information File 1, Figure S3). The lifetimes determined by single-exponential fitting were 1430 (**5a**) and 870 ps (**5b**), respectively (Table 1, entries 3 and 4). Double-exponential decay was, however, observed for the TEMPO-substituted NPBF derivatives **2a** and **2b**, where the lifetimes were 220 (57%) and 1370 ps (43%) for **2a**, and 390 (70%) and 890 ps (30%) for **2b** (Table 1, entries 1 and 2). For **2a** and **2b**, intermolecular charge transfer processes induced by the TEMPO moiety may account for the double-exponential decay curves to some extent.

**Figure 1:**
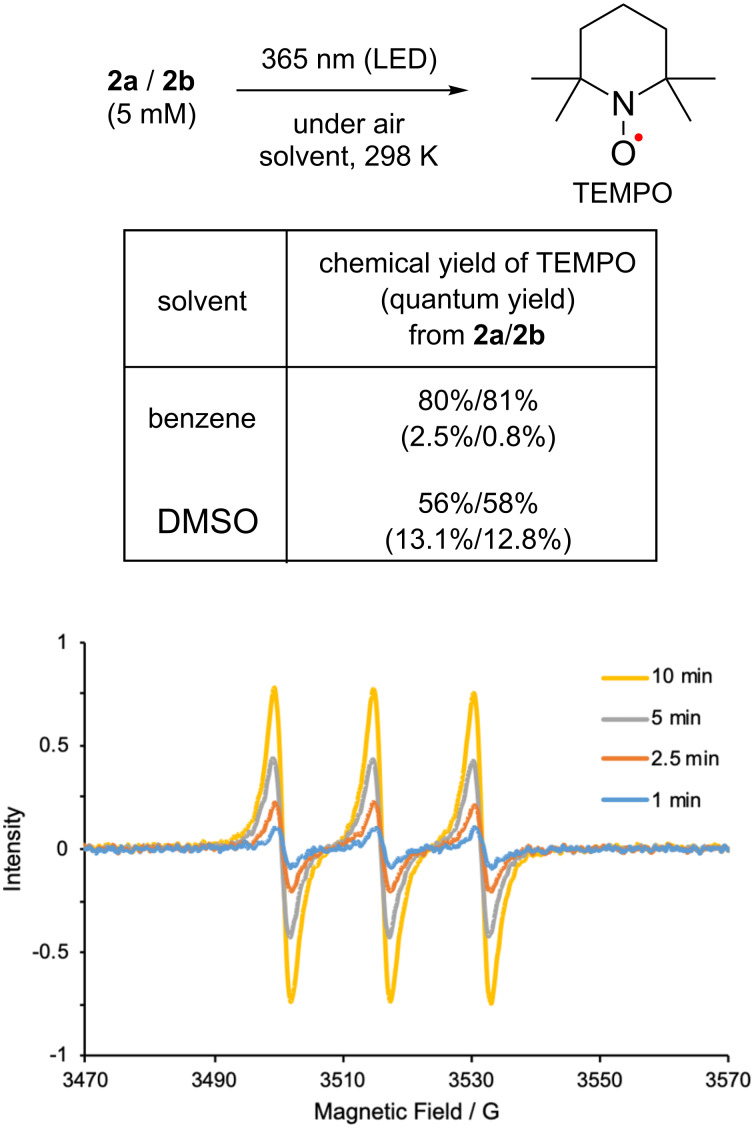
Photochemical generation of TEMPO from **2a** and **2b**. EPR spectra acquired during the photolysis of **2a** (5 mM) in benzene using 365 nm LED light under air atmosphere.

**Figure 2:**
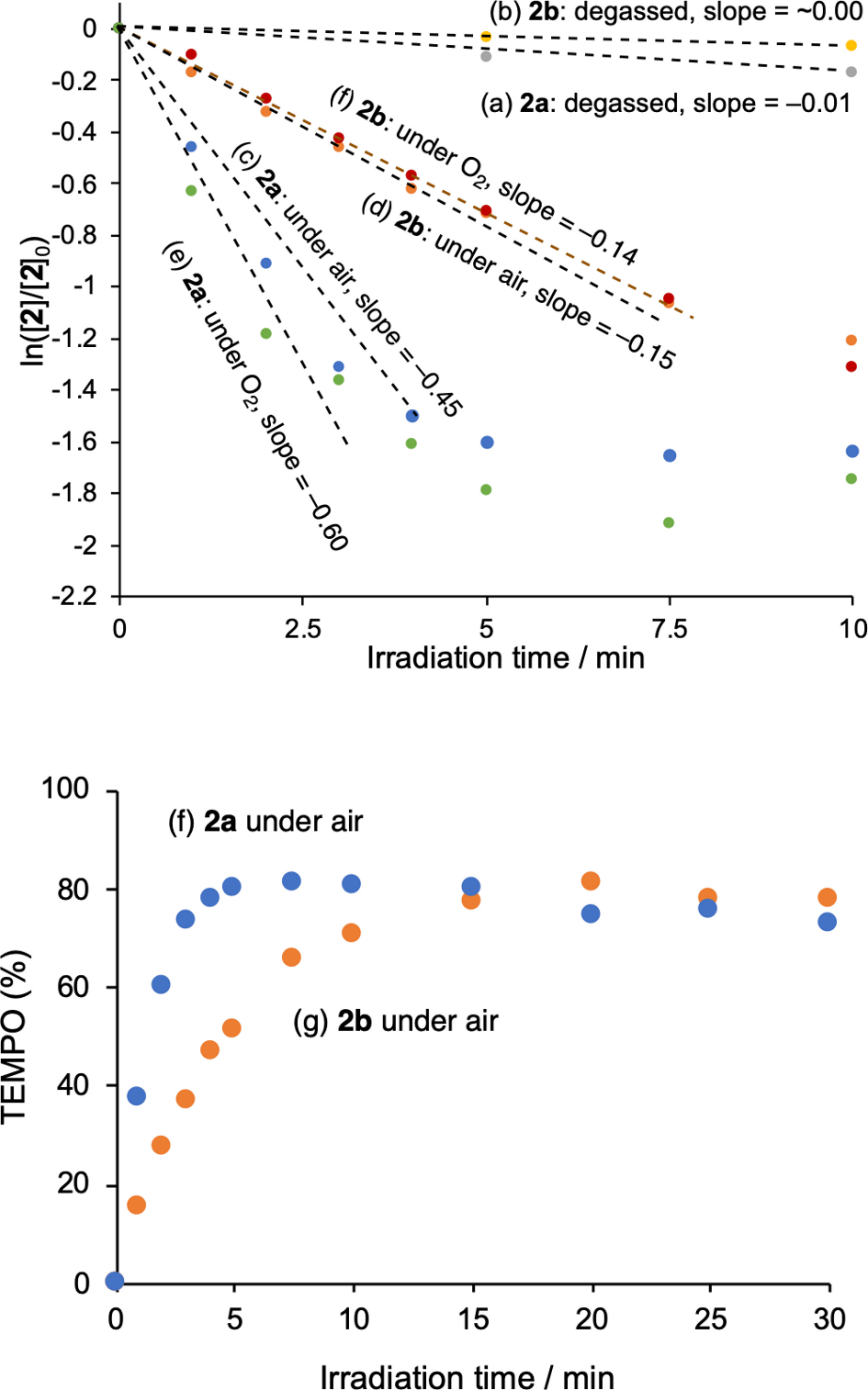
Time profile for photochemical generation of TEMPO radical from **2** (5 mM) at ≈298 K in benzene: (a) from **2a** under degassed conditions, (b) from **2b** under degassed conditions, (c,g) from **2a** under air conditions, (d,h) from **2b** under air conditions, (e) from **2a** under O_2_, (f) from **2b** under O_2_.

OP photolysis of **2a** (5 mM) was first conducted in benzene at ≈298 K using 365 nm light (6.02 × 10^15^ photons s^−1^) under atmospheric conditions (Figure 1). Clean release of the TEMPO radical was confirmed by measuring the electron paramagnetic resonance (EPR) signals of the typical nitroxide, *A*_N_ = 15.5 G (*g* = 2.00232, Figure 1 and Figure 2c). The first-order rate constant for generation of TEMPO in the bulk photoreaction was found to be *k* = 1.6 × 10^–5^ s^−1^. The amount of photochemically released TEMPO radical was determined by comparing the EPR intensity with the calibration curve of the standard TEMPO sample (Supporting Information File 1, Figure S4). The chemical yield of TEMPO was 80% after 10 min irradiation in benzene under air atmosphere (Figure 2g). Secondary photoreaction of TEMPO gradually decreased the chemical yield of TEMPO. The quantum yield (Φ) for photochemical release of the TEMPO radical was 2.5% at ≈1% conversion in the photolysis of **2a** in benzene under atmospheric conditions. Similar photochemical generation of the TEMPO radical was conducted with **2b** (5 mM, Supporting Information File 1, Figure S5 and Figure 2d,h). The clean generation of the TEMPO radical was also observed during photolysis under 365 nm irradiation in benzene at ≈298 K under atmospheric conditions, although the reaction was slower than that of **2a**, *k* = 5.5 x 10^–6^ s^–1^; Φ = 0.8% at ≈1% conversion of **2b**. However, the chemical yield of TEMPO was also high (81% after 20 min irradiation under the same conditions), although slow photochemical decomposition of TEMPO was observed with prolonged irradiation (Figure 2h). In DMSO, the quantum yield for the formation of TEMPO increased significantly to 13.1% (from **2a**) and 12.8% (from **2b**) at ≈1% conversion of **2** under atmospheric conditions (Figure 1). The notable effect of the solvent on the TEMPO generation may be due to the increase in the lifetime of the excited states. Photochemical decomposition of TEMPO in DMSO was found to be faster than that in benzene, but the chemical yield of TEMPO (56% from **2a** and 58% from **2b** after 40 s irradiation) was found to be lower than that obtained in benzene (Figure 1).

**Scheme 3:**
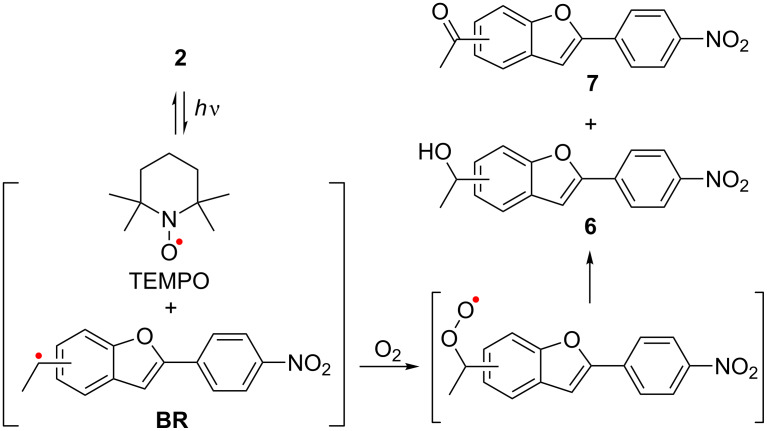
Photochemical generation of TEMPO radical and photoproducts **6** and **7** under air atmosphere.

To obtain insight into the mechanism of generation of the TEMPO radical, the photolysis of **2** was conducted under degassed conditions using the freeze-pump-thaw (FPT) method (Figure 2a,b). Interestingly, the generation of the TEMPO radical was highly suppressed under the photolysis conditions (Figure 2a,b). Under air conditions, however, the photochemical release of TEMPO was detected in benzene, as shown in Figure 2c,d. Faster formation of TEMPO was observed when O_2_ atmosphere was used instead of an air atmosphere (Figure 2e,f). Therefore, the O_2_ molecule may play an important role in clean generation of the TEMPO radical during photolysis. Indeed, the compounds oxidized at the benzylic carbon, **6** and **7**, were isolated in 15% (15%) and 56% (42%) yield in the photolysis of **2a** and **2b** under atmospheric conditions, respectively (Scheme 3), indicating that under degassed conditions, the photochemically generated radical pair returns to the starting compound **2** with rapid radical recombination. Over 70% of the caged TEMPO **2a** and ≈85% of **2b** were recovered after 2 h of irradiation under degassed conditions. The retarded formation of TEMPO after 5 min of irradiation is due to the decrease in the relative absorbance of **2a** to those of primary photoproducts (Figure 2c,e).

**Figure 3:**
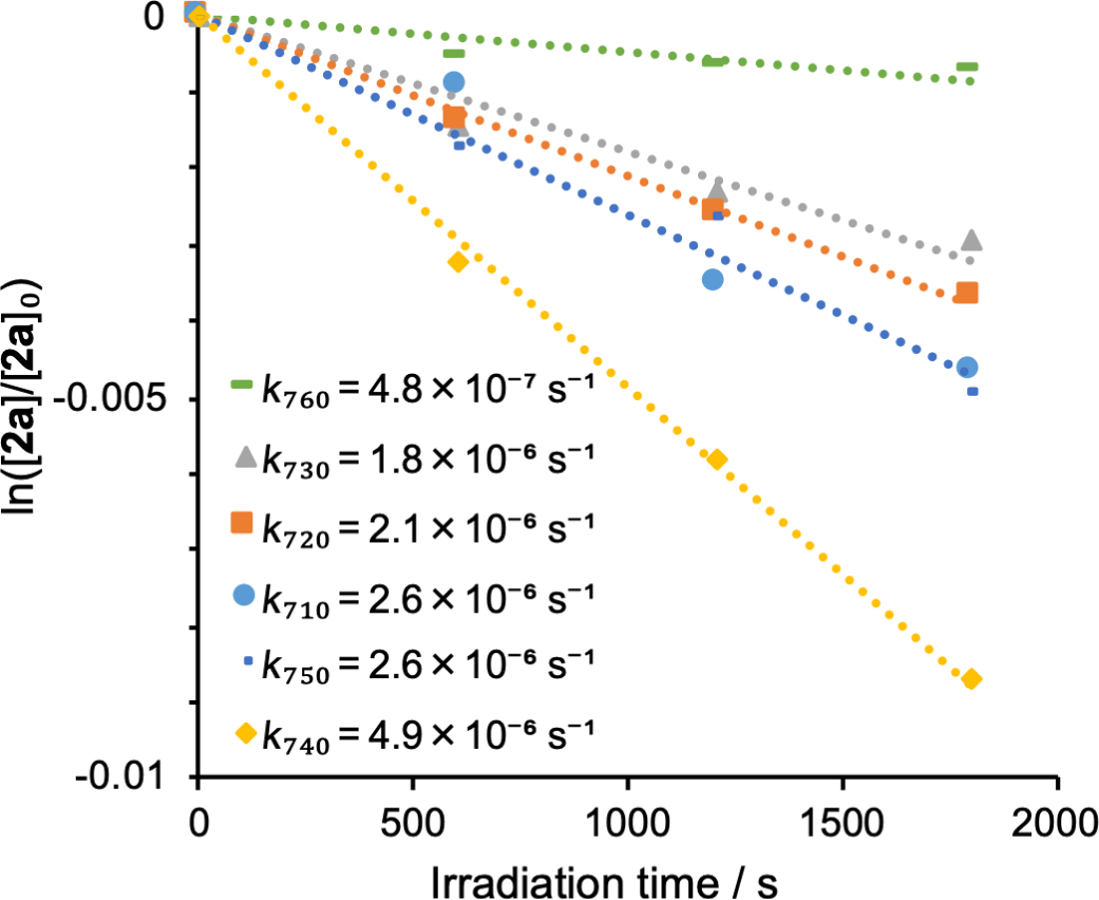
Time profile, ln([**2a**]/[**2a**]_0_) versus irradiation time, of two-photon uncaging reaction of TEMPO in the photolysis of **2a** in benzene, at wavelengths of 710–760 nm and power of 700 mW.

The TP photolysis of **2a** (10 mM) and **2b** (10 mM) was carried out in benzene under atmospheric conditions using 710, 720, 730, 740, 750, and 760 nm near infrared light from a Ti:sapphire laser (pulse width 100 fs, 80 MHz) emitting at an average of 700 mW (Figure 3 for **2a** and Supporting Information File 1, Figure S6 for **2b**). The typical EPR signals of TEMPO were also observed after TP excitation of **2a** and **2b** (Supporting Information File 1, Figure S7). The formation of TEMPO at 740 nm, *k*_740_ = 4.9 × 10^−6^ s^−1^ in the bulk photoreaction, was the fastest in the TP-uncaging reaction of **2a** (Figure 3). For the uncaging reaction of **2b**, the rate of consumption under 730 nm irradiation, *k*_730_ = 1.6 × 10^–6^ s^−1^, was larger than those at other wavelengths (Supporting Information File 1, Figure S6).

**Figure 4:**
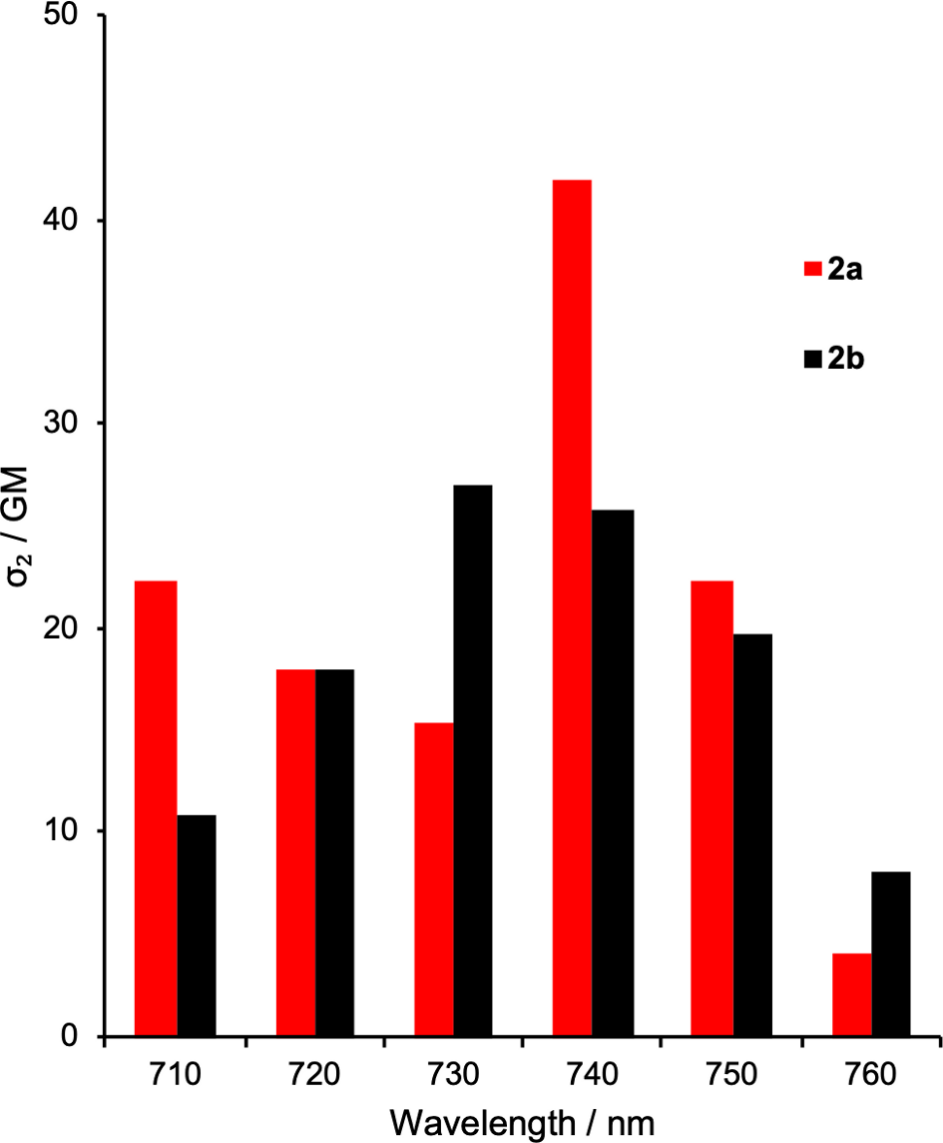
ESR spectra acquired during the photolysis of **2a** (5 mM) in benzene using 365 nm light.

The TP action spectra of **2a** and **2b** in benzene are shown in Figure 4, where the spectra were obtained by extrapolation from the absolute TP cross-section of the parent NPBF (18 GM) at 720 nm [58]. The TP cross-section of **2a** was higher than that of **2b** by ≈15 GM. This higher GM value may be due to the stronger donor–acceptor character of **2a** relative to that of **2b**, because the electron-donating alkyl group is located at the *para-*position of the *p*-nitrophenyl group in **2a**.

**Scheme 4:**
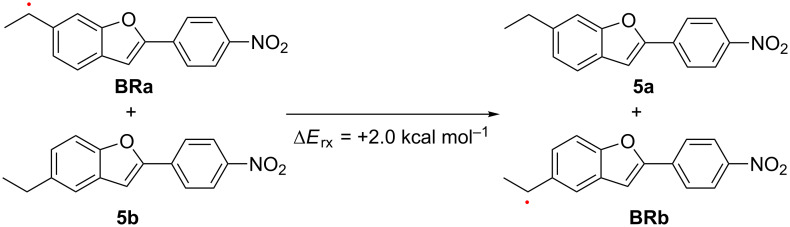
Isodesmic reaction from **BRa** and **5b** to **5a** and **BRb**.

As observed in the OP uncaging reaction at 365 nm, the efficiency of the TP-induced TEMPO uncaging reaction of **2a** was almost three times higher than that of **2b** in benzene. This is attributed to the substituent effect of the *meta*-alkoxy group on the reactivity in the electronically excited states [70]. Moreover, the relative stability of radicals **BRa** and **BRb** generated by the photolysis of **2a** and **2b** had an important impact on the uncaging efficiency. The isodesmic reaction shown in Scheme 4 suggests the radical **BRa** derived from **2a** was 2.04 kcal mol^−1^ more stable than **BRb** generated from **2b** based on DFT calculations at the B3LYP/6-31G(d) level of theory. In DMSO, no significant difference between **2a** and **2b** was observed for the photochemical release of the TEMPO species, although the solvent effect is not clearly explained.

As mentioned above, the spatiotemporally controlled generation of the radical pair of TEMPO and **BR** was confirmed in the photolysis of compounds **2a** and **2b**. Because free radicals play important roles as anticancer therapeutic agents, the cytocidal effect of the radical released from compound **2a** was also tested in vitro using lung cancer cells. One hundred thousand Lewis lung carcinoma (LLC) cells were seeded into 24-well plates (medium: DMEM) and incubated overnight at 37 °C under an atmosphere of 95% air and 5% CO_2_. The medium was replaced with fresh phenol-red free DMEM containing various concentrations of **2a** (0, 10, 100 μg mL^−1^) and further incubated for 4 h under the same conditions. Without exposure to light, **2a** itself exhibited slight cytotoxicity based on trypan blue exclusion, and ≈80–90% living cells remained in the medium containing of 100 μg mL^−1^ of **2a** (Supporting Information File 1, Figure S8).

**Figure 5:**
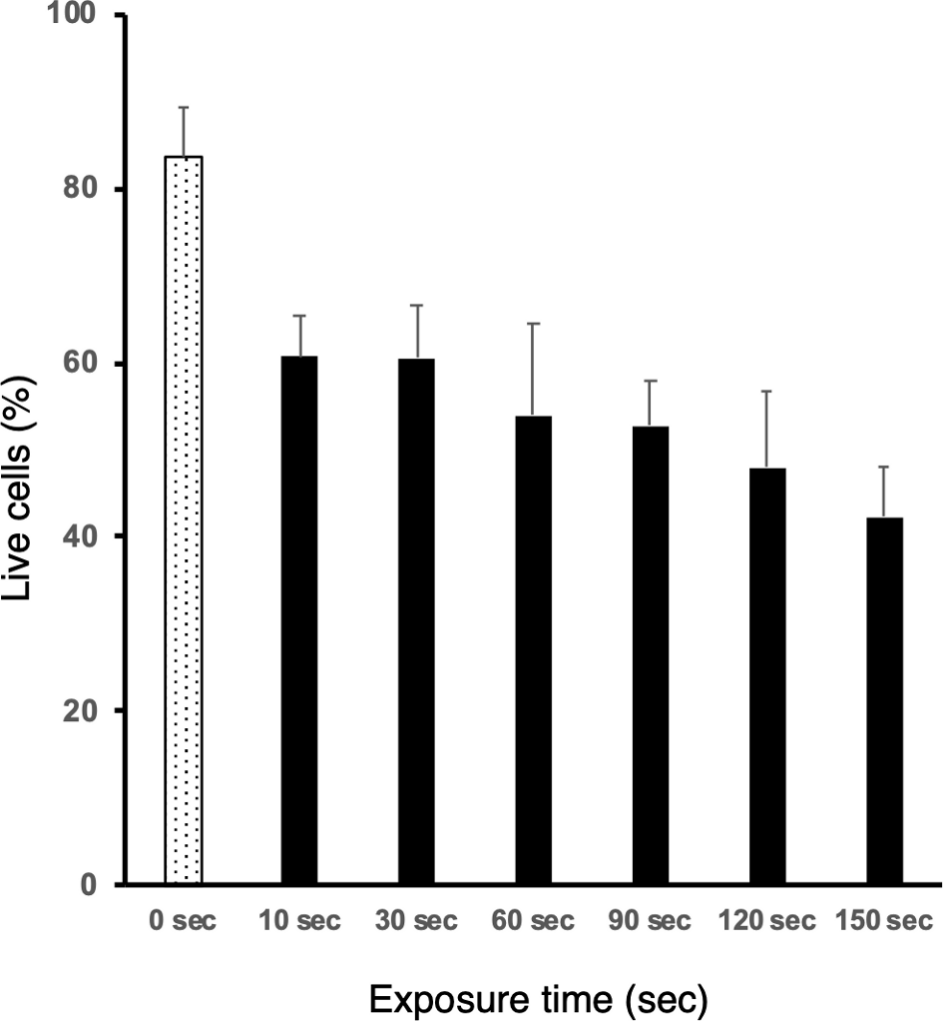
Irradiation time-dependent decline in viability of LLC cells with compound **2a**.

**Figure 6:**
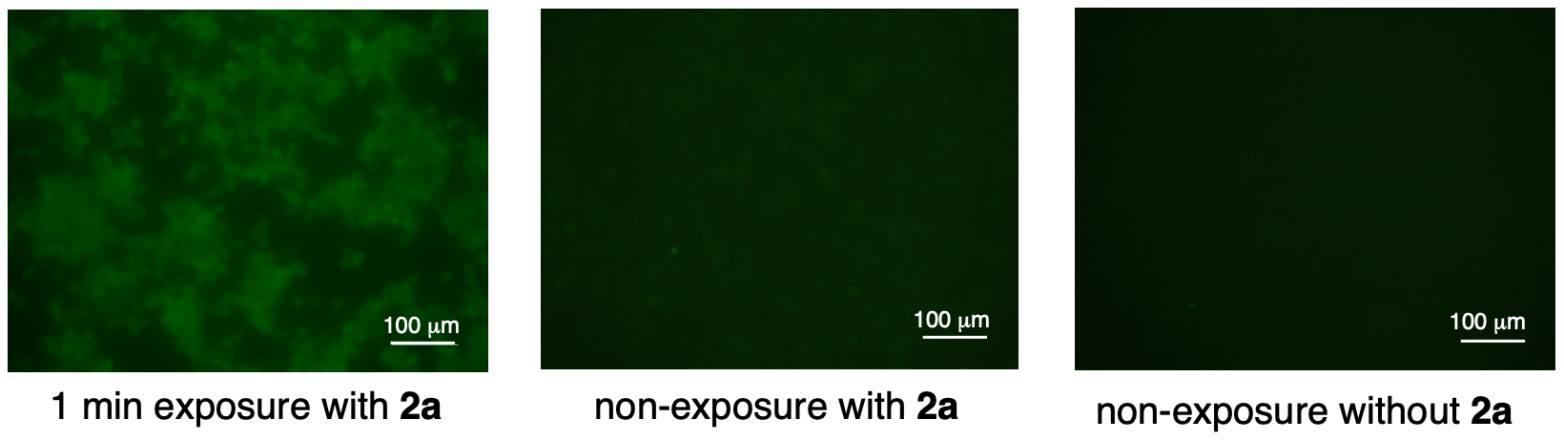
Detection of intracellular ROS only in irradiated LLC cells with **2a**-containing medium.

The cytocidal effect of the radicals released from compound **2a** on LLC cells was also tested. Four hours after 1 min exposure to 360 nm light in various concentrations of **2a**-containing medium, the number of living cells decreased in a **2a** concentration-dependent manner (Supporting Information File 1, Figure S9). After exposure of the cells in the medium containing 100 μg mL^−1^ of **2a**, the number of living cells decreased significantly compared to that without exposure (66.5% vs 87.8%, Supporting Information File 1, Figure S10). An irradiation-time-dependent decline in the viability of the LLC cells was also observed (Figure 5). To evaluate whether the cytocidal effect was due to photochemical radical generation, cells exposed to 360 nm light for 1 min and the unexposed congeners were stained by using a ROS-ID oxidative stress detection kit (Enzo Life Sciences, Farmingdale, NY, U.S.A.). Reactive oxygen species (ROS) were detected in the cells irradiated in the **2a**-containing medium, but not in the non-irradiated cells in **2a**-containing medium or the irradiated cells without **2a**-containing medium (Figure 6). Thus, the preliminary analyses indicated that the photochemical generation of radicals from **2a** induced cancer cell death in vitro, although no in vivo study was performed because of the low water solubility of **2a**. At this point, we cannot rule out generate of ROS by photosensitization of the chromophore in the presence of O_2_ for the cytotoxicity.

## Conclusion

In the present study, novel caged nitroxides **2a** and **2b** having a TP-responsive chromophore were synthesized, and OP- and TP-induced generation of the TEMPO radical with these species was examined. The quantum yields for generation of the TEMPO radical from **2a** and **2b** were determined to be 2.5% and 0.8% in benzene, respectively. The quantum yields in DMSO were found to be higher than those in benzene, 13.1% and 12.8%, respectively. The OP-uncaging efficiency (ε × Φ) was found to be 480 and 175 for **2a** and **2b**, respectively, at 360 ± 10 nm, in benzene, and 3026 and 2995 in DMSO, respectively. The TP efficiency of the TEMPO uncaging reaction was found to be 1.1 GM at 740 nm for **2a** and 0.22 GM at 730 nm for **2b** in benzene. The TP-induced clean release of the TEMPO radical is expected to be applicable to further physiological studies and site-selective polymerization reactions.

## Experimental

All reagents were purchased from commercial sources and were used without additional purification, unless otherwise mentioned. Caged nitroxides **2a** and **2b** were prepared according to the methods described previously (Scheme 2) and were isolated by silica gel column chromatography and GPC. ^1^H and ^13^C NMR spectra were reported in parts per million (δ) by using CDCl_3_. IR spectra were recorded with a FTIR spectrometer. UV–vis spectra were taken by a SHIMADZU UV-3600 Plus spectrophotometer. Mass spectra were measured by a Mass Spectrometric Thermo Fisher Scientific LTQ Orbitrap XL, performed by the Natural Science Center for Basic Research and Development (N-BARD), Hiroshima University.

### Preparation of caged compounds **2a** and **2b**

**6-Ethyl-2-(4-nitrophenyl)benzofuran (5a).** 4-Nitro-1-iodobenzene (16.3 g, 65.5 mmol), Pd(dppf)Cl_2_ (0.97 g, 1.3 mmol), PPh_3_ (recrystallized, 0.51 g, 1.9 mmol) and CuI (0.25 g, 1.3 mmol) were added under N_2_ atmosphere followed by toluene (97 mL) and iPr_2_NH (49 mL, 359 mmol). The mixture was stirred for 10 min, and TMSA (11.5 mL, 81.6 mmol) in toluene (64 mL) was added at room temperature. It was stirred until all iodobenzene was consumed (20 min). TBAF 1 M in THF (100 mL, 100 mmol) was added followed by 5-ethyl-2-iodophenol (21.6 g, 86.9 mmol). The temperature was increased to 80 °C, and the mixture was stirred for 21.5 h. The reaction was quenched with 10% aqueous citric acid (400 mL) and extracted with DCM. The combined extracts were washed with 10% aqueous NaOH (400 mL), water and dried with anhydrous MgSO_4_. The solvent was removed by rotary evaporation and the crude product was purified by silica gel column chromatography (hexane/EtOAc 10:1, v/v) to give 6-ethyl-2-(4-nitrophenyl)benzofuran (**5a**, 10.0 g, 57.3%). mp 114 °C; IR (KBr, cm^−1^): 3429, 2968, 1601, 1520, 1344, 1108, 828, 825, 754, 692; ^1^H NMR (400 MHz, CDCl_3_) δ (ppm) 8.30 (d, *J* = 9.0 Hz, 2H), 7.98(d, *J* = 9.0 Hz, 2H), 7.54 (d, *J* = 8.0 Hz, 1H), 7.39 (s, 1H), 7.20 (d, *J* = 0.9 Hz, 1H), 7.14 (dd, *J* = 8.0, 1.4 Hz, 1H), 2.80 (q, *J* = 7.6 Hz, 2H), 1.32 (t, *J* = 7.6 Hz, 3H); ^13^C NMR (100 MHz, CDCl_3_) δ (ppm) 156.05 (C), 152.86 (C), 147.12 (C), 143.06 (C), 136.56 (C), 126.43 (C), 125.01 (CH), 124.33 (CH), 124.07 (CH), 121.22 (CH), 110.42 (CH), 105.12 (CH), 29.25 (CH_2_), 15.84 (CH_3_); HRMS–ESI (*m*/*z*): [M^−^] calcd. for C_16_H_13_NO_3_, 267.09009; found, 267.09064.

**2,2,6,6-Tetramethyl-1-(1-(2-(4-nitrophenyl)benzofuran-6-yl)ethoxy)piperidine (2a).** Under air, TEMPO (0.23 g, 1.5 mmol), 6-ethyl-2-(4-nitrophenyl)benzofuran (**5a**, 1.26 g, 4.72 mmol), Cu(OAc)_2_ (16.5 mg, 0.092 mmol), bpy (13.8 mg, 0.094 mmol), TBHP (aqueous 70%, 0.41 mL, 2.9 mmol) were added into a two-necked flask in the dark. The reaction was stirred at 60 °C for 15 h. Upon completion, the mixture was purified silica gel column chromatography (hexane/ether 15:1, v/v) to give **2a** (236 mg, 37.8%). mp 109 °C; IR (KBr, cm^−1^): 3429, 2934, 1600, 1521, 1345, 1062, 825; ^1^H NMR (400 MHz, CDCl_3_) δ (ppm) 8.31 (d, *J* = 9.0 Hz, 2H), 7.99 (d, *J* = 9.0 Hz, 2H), 7.57 (d, *J* = 8.1 Hz, 1H), 7.54 (s, 1H), 7.25 (dd, *J* = 8.1, 1.2 Hz, 1H), 7.21 (d, *J* = 0.7 Hz, 1H), 4.91 (q, *J* = 6.7 Hz, 1H), 1.54 (d, *J* = 6.6 Hz, 3H), 1.51 (s, 3H), 1.32 (s, 6H), 1.20 (s, 3H), 1.05 (s, 3H), 0.66 (s, 3H); ^13^C NMR (100 MHz, CDCl_3_) δ (ppm) 155.69 (C), 153.25 (C), 147.18 (C), 144.70 (C), 136.50 (C), 127.39 (C), 125.08 (CH), 124.33 (CH), 122.65 (CH), 121.04 (CH), 109.47 (CH), 105.13 (CH), 83.28 (CH), 59.79 (C), 40.43 (CH_2_), 34.23 (CH_3_) , 23.82 (CH_2_), 20.40 (CH_3_), 17.24 (CH3); HRMS–ESI (*m*/*z*): [M + H]^+^ calcd. for C_25_H_30_N_2_O_4_, 423.22783; found, 423.22754.

**5-Ethyl-2-(4-nitrophenyl)benzofuran (5b).** 4-Nitro-1-iodobenzene (16.8 g, 67.5 mmol), Pd(dppf)Cl_2_ (1.0 g, 1.3 mmol), PPh_3_ (recrystallized, 0.53 g, 2.0 mmol) and CuI (0.26 g, 1.3 mmol) were added under N_2_ atmosphere followed by toluene (100 mL) and iPr_2_NH (50.5 mL, 370 mmol). The mixture was stirred for 10 min, and TMSA (1.75 mL, 12.5 mmol) in toluene (10 mL) was added at room temperature. It was stirred until all iodobenzene was consumed (20 min). TBAF 1 M in THF (100 mL, 100 mmol) was added followed by 4-ethyl-2-iodophenol (22.22 g, 89.6 mmol). The temperature was increased to 80 °C, and the mixture was stirred for 20 h. The reaction was quenched with 10% aqueous citric acid (666 mL) and extracted with DCM. Combined extracts were washed with 10% aqueous NaOH (666 mL), water and dried with anhydrous MgSO_4_. The solvent was removed by rotary evaporation and the crude product was purified silica gel column chromatography (hexane/EtOAc 10:1, v/v) to give 5-ethyl-2-(4-nitrophenyl)benzofuran (**5b**, 12.2 g, 67.6%). mp 129 °C; IR (KBr, cm^−1^): 2922, 1601, 1514, 1340, 1194, 853, 811, 754, 690; ^1^H NMR (400 MHz, CDCl_3_) δ (ppm) 8.30 (d, *J* = 9.0 Hz, 2H), 7.99 (d, *J* = 9.0 Hz, 2H), 7.46 (d, *J* = 9.2 Hz, 1H), 7.44 (s, 1H), 7.21 (dd, *J* = 8.4, 1.8 Hz, 1H), 7.19 (d, *J* = 0.8 Hz, 1H), 2.76 (q, *J* = 7.6 Hz, 2H) 1.30 (t, *J* = 7.6 Hz, 3H); ^13^C NMR (100 MHz, CDCl_3_) δ (ppm) 154.12 (C), 153.38 (C), 147.21 (C), 139.74 (C), 136.47 (C), 128.79 (C), 126.24 (CH), 125.12 (CH), 124.29 (CH), 120.14 (CH), 111.12 (CH), 105.04 (CH), 28.83 (CH_2_), 16.15 (CH_3_); HRMS–ESI (*m*/*z*): [M^−^] calcd. for C_16_H_13_NO_3_, 267.09009; found, 267.09030.

**2,2,6,6-Tetramethyl-1-(1-(2-(4-nitrophenyl)benzofuran-5-yl)ethoxy)piperidine (2b).** Under air, TEMPO (46.8 mg, 0.3 mmol), 5-ethyl-2-(4-nitrophenyl)benzofuran (**5b**, 267 mg, 1 mmol), Cu(OAc)_2_ (3.6 mg, 0.02 mmol), bpy (3.1 mg, 0.02 mmol), TBHP (aqueous 70%, 0.086 mL, 0.6 mmol) were added into a Schlenk tube in the dark. The reaction was stirred at 60 °C for 16.5 h. Upon completion, the mixture was purified by silica gel column chromatography (hexane/ether 15:1, v/v) to give **2b** (66 mg, 52%). mp 144 °C; IR (KBr, cm^−1^): 2922, 1602, 1520, 1342, 1108, 852, 746; ^1^H NMR (400 MHz, CDCl_3_) δ (ppm) 8.31 (d, *J* = 9.0 Hz, 2H), 7.99 (d, *J* = 9.0 Hz, 2H), 7.57 (d, *J* = 1.6 Hz, 1H), 7.50 (d, *J* = 8.6 Hz, 1H), 7.36 (dd, *J* = 8.6, 1.7 Hz, 1H), 7.23 (s, 1H), 4.88 (q, *J* = 6.6 Hz, 1H), 1.53 (d, *J* = 6.7 Hz, 3H), 1.51 (s, 3H), 1.33 (s, 6H), 1.19 (s, 3H), 1.02 (s, 3H), 0.60 (s, 3H); ^13^C NMR (100 MHz, CDCl_3_) δ (ppm) 154.75 (C), 153.43 (C), 147.24 (C), 141.55 (C), 136.47 (C), 128.34 (C), 125.15 (CH), 125.04 (CH), 124.32 (CH), 119.50 (CH), 110.98 (CH), 105.39 (CH), 83.13 (CH), 59.76 (C), 40.42 (CH_2_), 34.37 (CH_3_), 23.78 (CH_2_), 20.38 (CH_3_), 17.24 (CH_3_); HRMS–ESI (*m*/*z*): [M + H]^+^ calcd. for C_25_H_30_N_2_O_4_; 423.22783; found 423.22757.

**1-(2-(4-Nitrophenyl)benzofuran-6-yl)ethan-1-ol (6a).** mp 133 °C; ^1^H NMR (400 MHz, CDCl_3_) δ (ppm) 8.31 (d, *J* = 9.0 Hz, 2H), 7.99 (d, *J* = 9.0 Hz, 2H), 7.61 (d, *J* = 7.7 Hz, 1H), 7.61 (s, 1H), 7.30 (dd, *J* = 8.1, 1.3 Hz, 1H), 7.22 (s, 1H), 5.06 (m, 1H), 1.90 (d, *J* = 3.6 Hz, 1H), 1.57 (d, *J* = 6.5 Hz, 3H); ^13^C NMR (100 MHz, CDCl_3_) δ (ppm) 155.72 (C), 153.61 (C), 147.24 (C), 144.47 (C), 136.26 (C), 127.96 (C), 125.16 (CH), 124.34 (CH), 121.56 (CH), 121.34 (CH), 108.33 (CH), 104.97 (CH), 70.50 (CH), 25.58 (CH_3_).

**1-(2-(4-Nitrophenyl)benzofuran-5-yl)ethan-1-ol (6b).** mp 149 °C; ^1^H NMR (400 MHz, CDCl_3_) δ (ppm) 8.30 (d, *J* = 9.0 Hz, 2H), 7.99 (d, *J* = 9.0 Hz, 2H), 7.66 (s, 1H), 7.53 (d, *J* = 8.5 Hz, 1H), 7.39 (dd, *J* = 8.5, 1.7 Hz, 1H), 7.22 (s, 1H), 5.04 (q, *J* = 6.4 Hz, 1H), 1.88 (s, 1H), 1.57 (d, *J* = 6.4 Hz, 3H); ^13^C NMR (100 MHz, CDCl_3_) δ (ppm) 154.93 (C), 153.81 (C), 147.29 (C), 141.48 (C), 136.22 (C), 128.74 (C), 125.24 (CH), 124.32 (CH), 123.67 (CH), 118.28 (CH), 111.45 (CH), 105.14 (CH), 70.47 (CH), 25.64 (CH_3_); HRMS–ESI (*m*/*z*): [M^−^] calcd. for C_16_H_13_NO_4_, 283.08501; found, 283.08548.

**1-(2-(4-Nitrophenyl)benzofuran-6-yl)ethan-1-one (7a).** mp 214 °C; ^1^H NMR (400 MHz, CDCl_3_) δ (ppm) 8.35 (d, *J* = 8.9 Hz, 2H), 8.18 (s, 1H), 8.05 (d, *J* = 8.9 Hz, 1H), 7.93 (dd, *J* = 8.2, 1.3 Hz, 1H), 7.71 (d, *J* = 8.2 Hz, 1H), 7.29 (s, 1H), 2.70 (s, 3H); ^13^C NMR (100 MHz, CDCl_3_) δ (ppm) 197.22 (C), 156.45 (C), 155.12 (C), 147.82 (C), 135.52 (C), 134.84 (C), 132.93 (C), 125.76 (CH), 124.41 (CH), 123.88 (CH), 121.41 (CH), 111.79 (CH), 104.85 (CH), 26.86 (CH_3_).

**1-(2-(4-Nitrophenyl)benzofuran-5-yl)ethan-1-one (7b).** mp 229 °C; ^1^H NMR (400 MHz, CDCl_3_) δ (ppm) 8.34 (d, *J* = 9.0 Hz, 2H), 8.30 (s, 1H), 8.04 (d, *J* = 8.6 Hz, 1H), 8.03 (d, *J* = 9.0 Hz, 2H), 7.62 (d, *J* = 8.7, 1H), 7.32 (s, 1H), 2.69 (s, 3H); ^13^C NMR (100 MHz, CDCl_3_) δ (ppm) 197.35 (C), 157.84 (C), 154.86 (C), 147.63 (C), 135.59 (C), 133.41 (C), 128.79 (C), 126.38 (CH), 125.51 (CH), 124.40 (CH), 122.86 (CH), 111.57 (CH), 105.38 (CH), 26.82 (CH_3_); HRMS–ESI (*m*/*z*): [M^−^] calcd. for C_16_H_11_NO_4_, 281.06936; found, 281.06970.

### Photoirradiation of LLC cells with compound **2a**

One hundred thousand Lewis lung carcinoma (LLC) cells were seeded into a 24-well plate (medium: DMEM) and incubated overnight at 37 °C in an atmosphere of 95% air and 5% CO_2_. The medium was replaced with fresh phenol-red free DMEM containing 100 µg/mL of compound **2a**. Four hours after various irradiation time of 360 nm light (0, 10, 30, 60, 90, 120, and 150 s) using a fluorescence microscope (BIOREVO BZ-9000, Keyence, Osaka, Japan), cell viability was determined by trypan blue exclusion. Bars represent the mean ± standard deviation (*n* = 4).

### Detection of intracellular ROS in irradiated LLC cells with **2a**-containing medium

Fifty thousand Lewis lung carcinoma (LLC) cells were seeded into 24-well plate (medium: DMEM) and incubated overnight at 37 °C in an atmosphere of 95% air and 5% CO_2_. The medium was replaced with fresh phenol-red free DMEM containing 0 or 100 µg/mL of compound **2a**. Thirty minutes after 1 min or no exposure of 360 nm light using a fluorescence microscope (BIOREVO BZ-9000, Keyence, Osaka, Japan), intracellular ROS were detected using the ROS-ID Oxidative Stress Detection Kit (Enzo Life Sciences, Farmingdale, NY, USA) in conjunction with fluorescence microscopy. Intracellular ROS was detected in the form of green fluorescence signals. Bars, 100 µm.

## Supporting Information

File 1 ^1^H and ^13^C NMR charts for new compounds and Figures S1–S8.
