# Supplementary material for: Photochemical generation of the 2,2,6,6-tetramethylpiperidine-1-oxyl (TEMPO) radical from caged nitroxides by near-infrared two-photon irradiation and its cytocidal effect on lung cancer cells
Source: Beilstein J Org Chem. 2019 Apr 10;15:863–73. doi: 10.3762/bjoc.15.84 (PMC6466695; doi:10.3762/bjoc.15.84)
Supplement: File 1 — 1H and 13C NMR charts for new compounds and Figures S1–S8. [file Beilstein_J_Org_Chem-15-863-s001.pdf]

BEILSTEIN JOURNAL OF ORGANIC CHEMISTRY

Supporting Information

for

Photochemical generation of the 2,2,6,6-tetramethylpiperidine-1-oxyl (TEMPO) radical from caged nitroxides by near-infrared two-photon irradiation and its cytocidal effect on lung cancer cells

Ayato Yamada, Manabu Abe, Yoshinobu Nishimura, Shoji Ishizaka, Masashi Namba, Taku Nakashima, Kiyofumi Shimoji and Noboru Hattori

Beilstein J. Org. Chem. **2019,** *15,* 863–873. doi:10.3762/bjoc.15.84

^{1}H and ^{13}C NMR charts for new compounds and Figures S1–S8

License and Terms: This is a supporting information file under the terms of the Creative Commons Attribution License (http://creativecommons.org/licenses/by/4.0). Please note that the reuse, redistribution and reproduction in particular requires that the authors and source are credited.
The license is subject to the *Beilstein Journal of Organic Chemistry* terms and conditions: (https://www.beilstein-journals.org/bjoc)

Experimental procedures, NMR spectra and photochemical properties data of all the compounds

Table of contents

	Page
^{1}H and ^{13}C spectra of compound **2**, **5**, **6**, and **7**	S3–10
UV–vis absorption spectra of compound **2a**, **2b**, **5a** and **5b**	S11
The thermal stability of **2a** and **2b**	S12
The charge transfer transition of **5a**	S12
The fluorescence lifetime of **2** and **5**	S13
Calibration curve of TEMPO	S14
OP uncaging reaction of **2b**	S14
TP uncaging reaction of **2b**	S15
The typical EPR signals by the TP excitation	S15
Confirmation of cytotoxicity by caged radical compounds	S16–S17

^{1}H and ^{13}C spectra of compounds

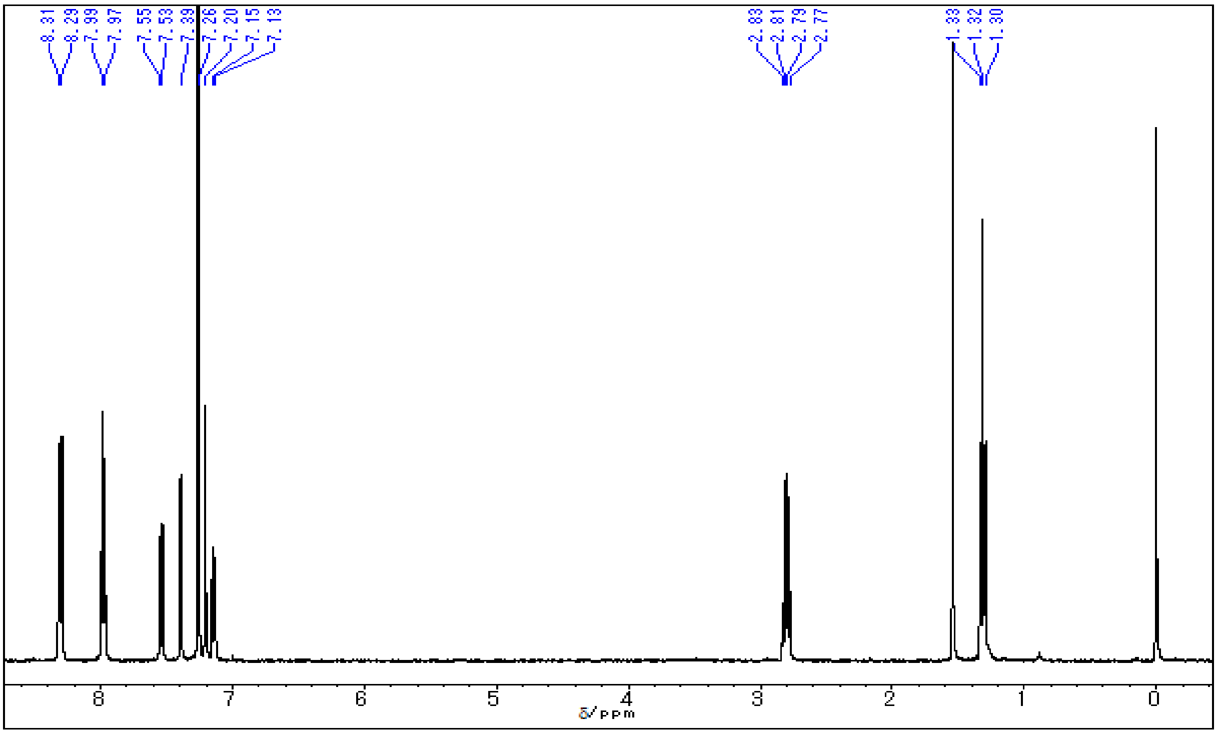

^{1}H NMR spectrum of **5a** (400 MHz, $CDCl_3$).

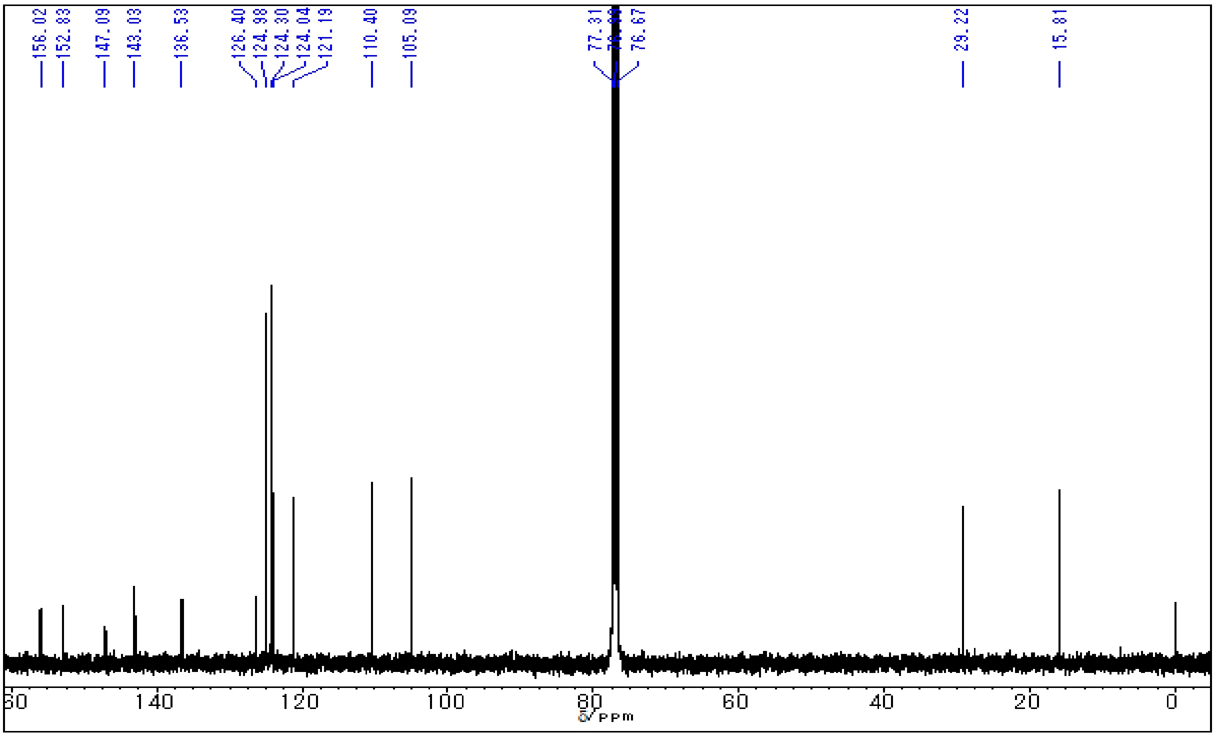

^{13}C NMR spectrum of **5a** (100 MHz, $CDCl_3$).

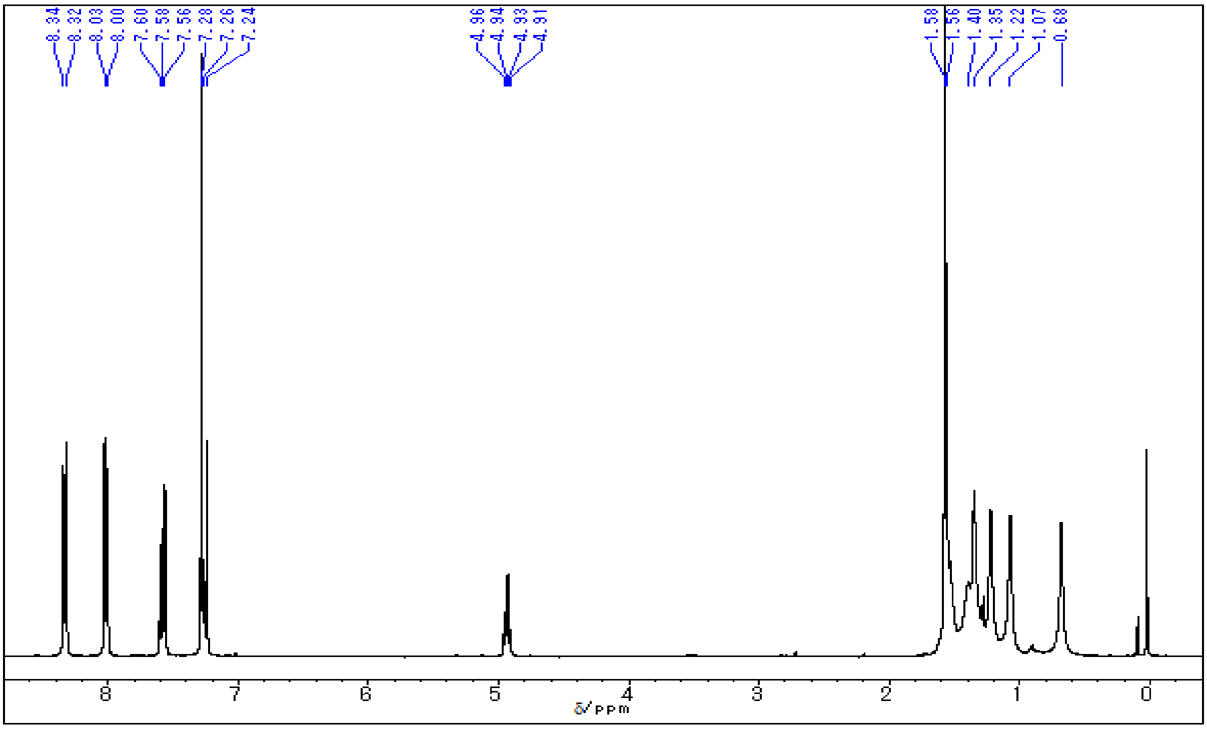

^{1}H NMR spectrum of **2a** (400 MHz, $CDCl_3$).

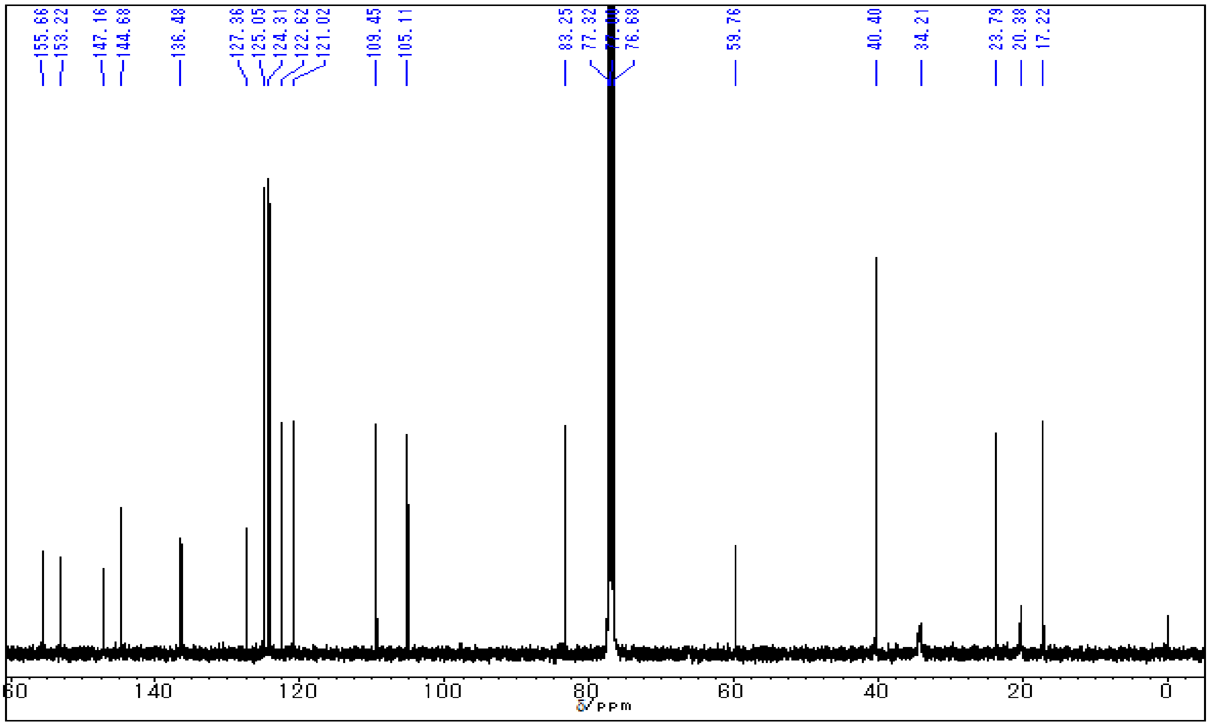

^{13}C NMR spectrum of **2a** (100 MHz, $CDCl_3$).

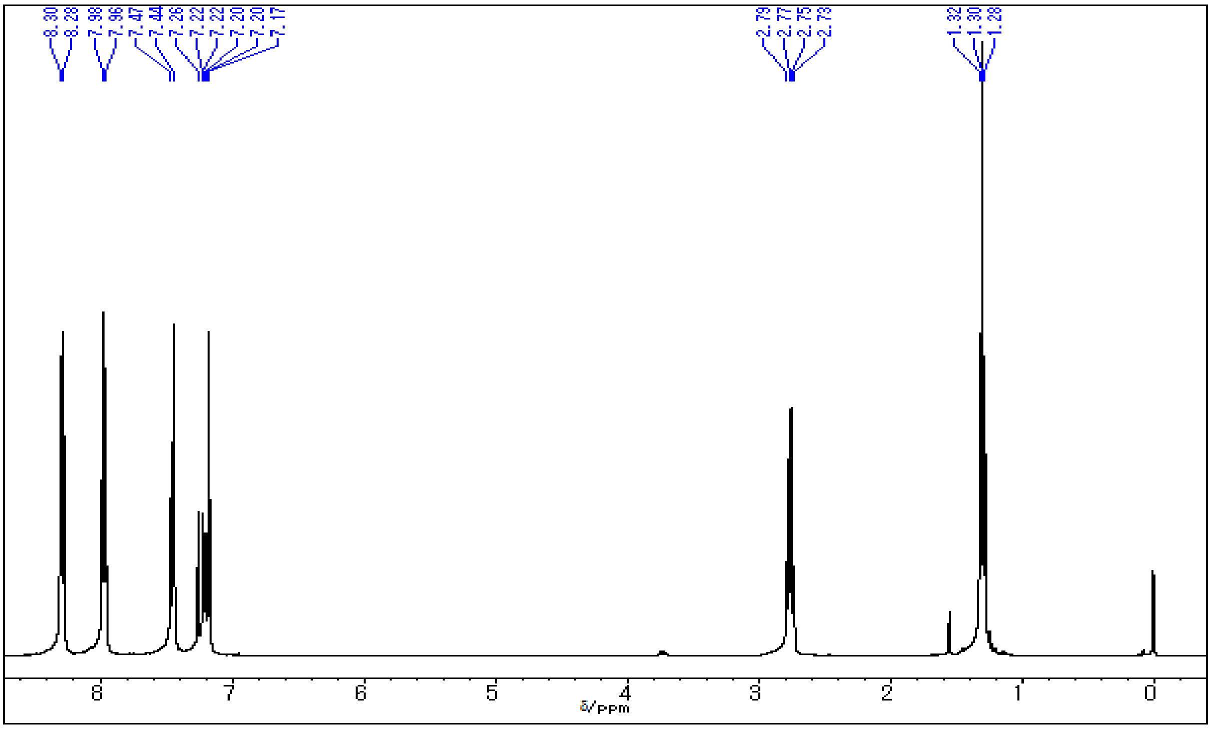

^{1}H NMR spectrum of **5b** (400 MHz, $CDCl_3$).

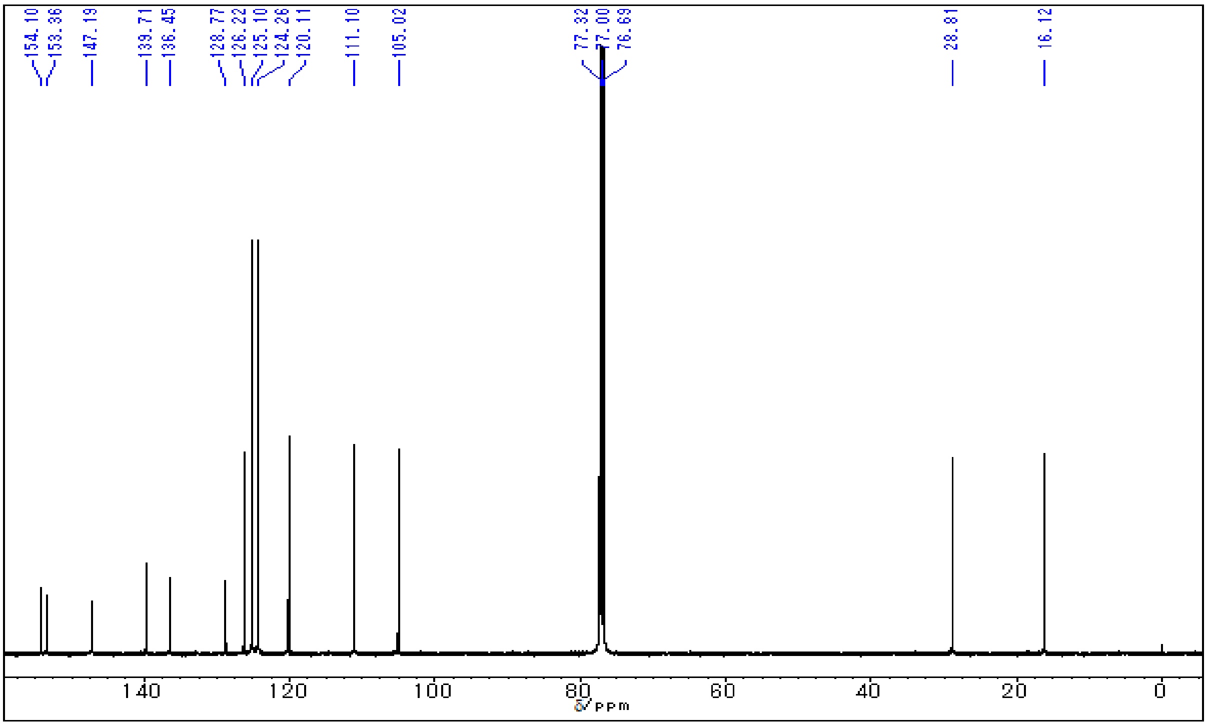

^{13}C NMR spectrum of **5b** (100 MHz, $CDCl_3$).

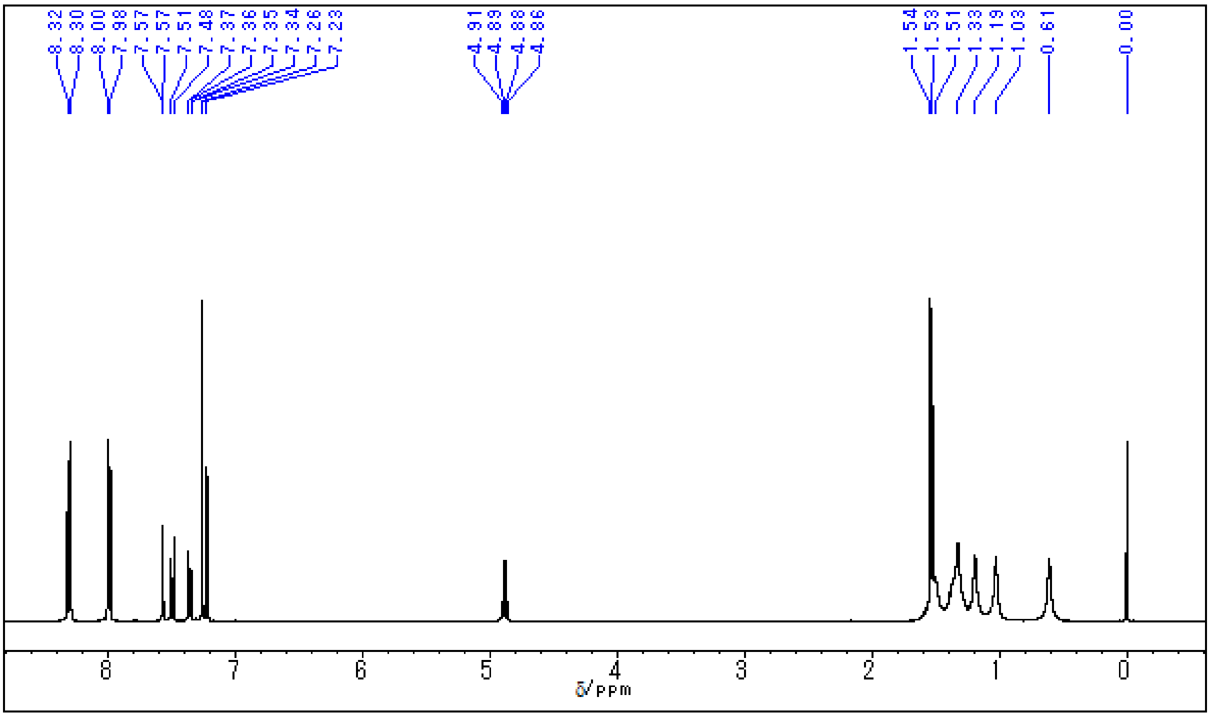

^{1}H NMR spectrum of **2b** (400 MHz, $CDCl_3$).

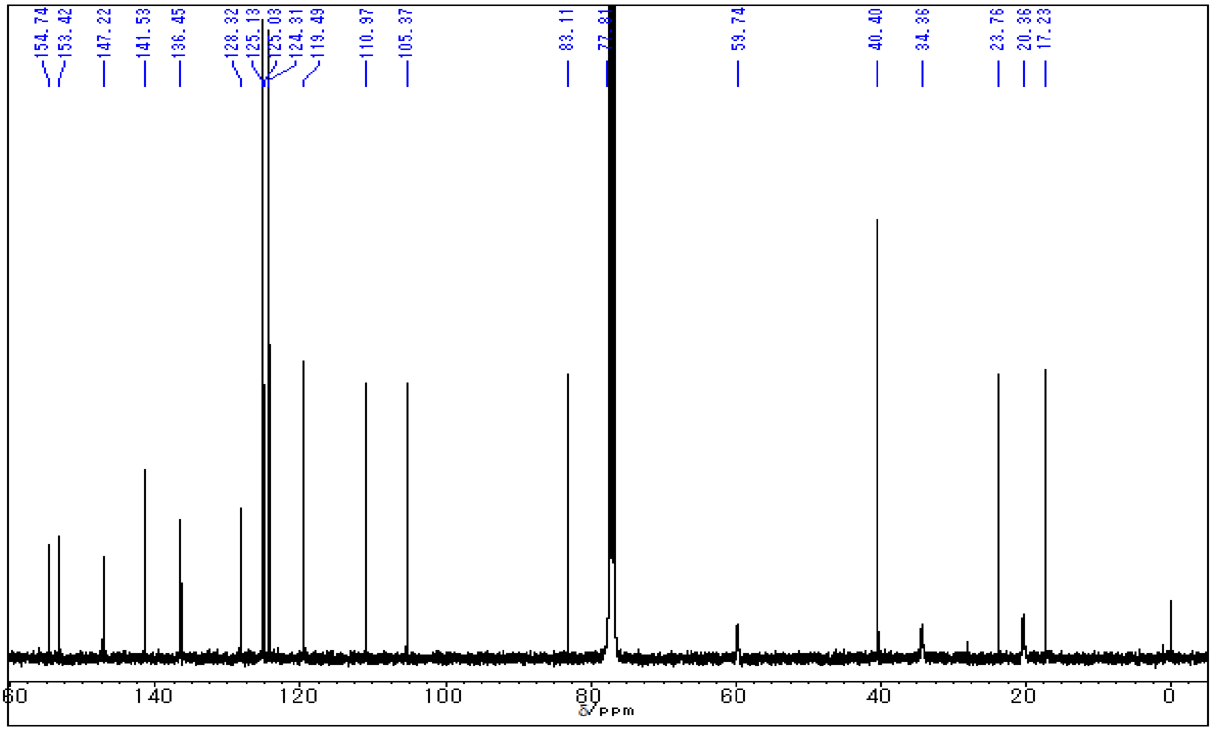

^{13}C NMR spectrum of **2b** (100 MHz, $CDCl_3$).

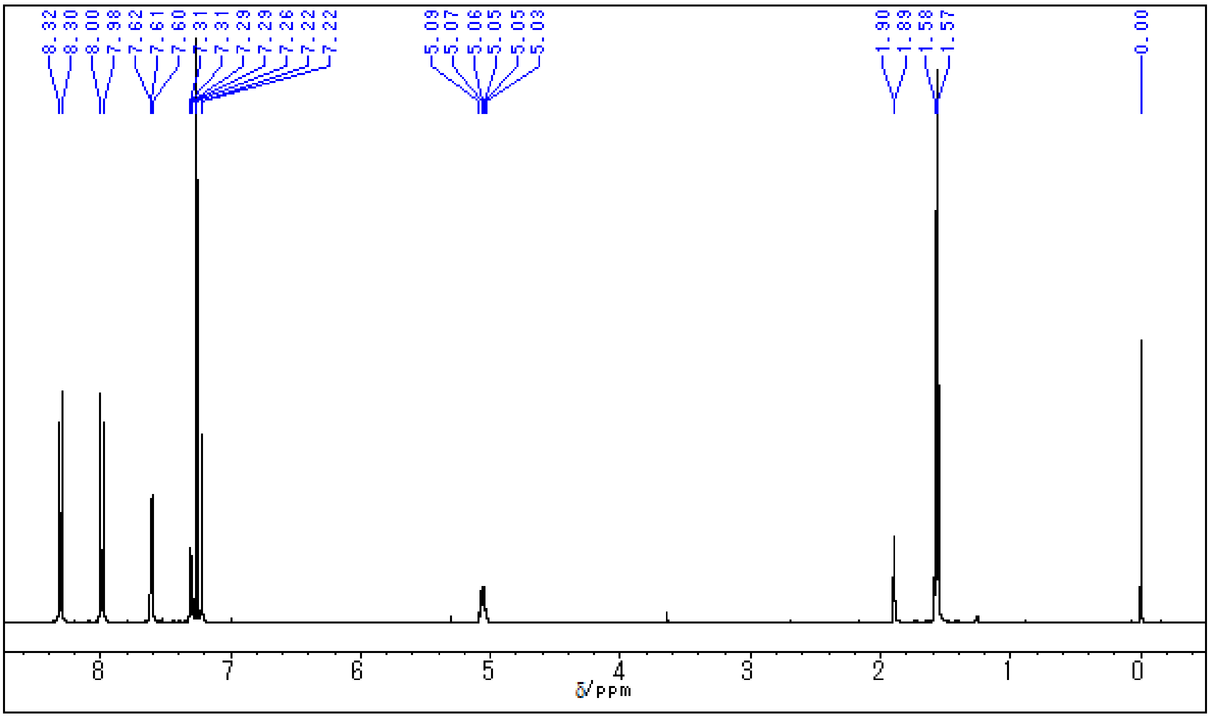

^{1}H NMR spectrum of **6a** (400 MHz, $CDCl_3$).

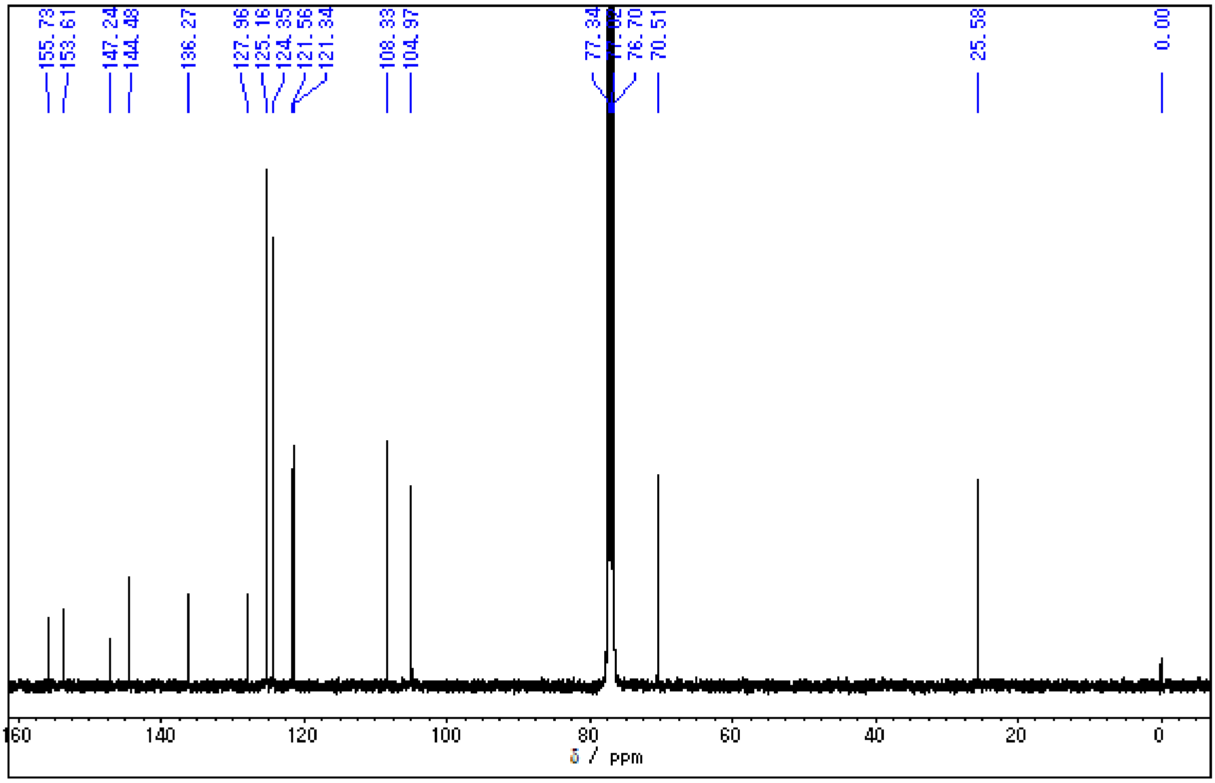

^{13}C NMR spectrum of **6a** (100 MHz, $CDCl_3$).

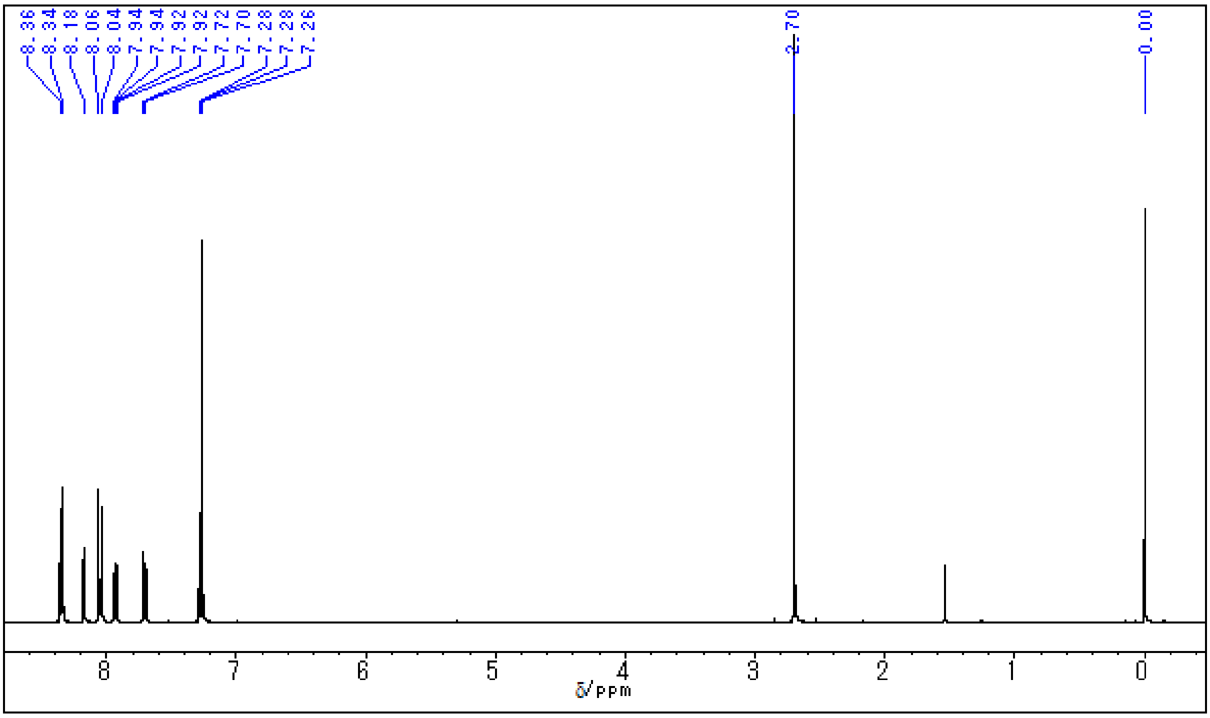

^{1}H NMR spectrum of **7a** (400 MHz, $CDCl_3$).

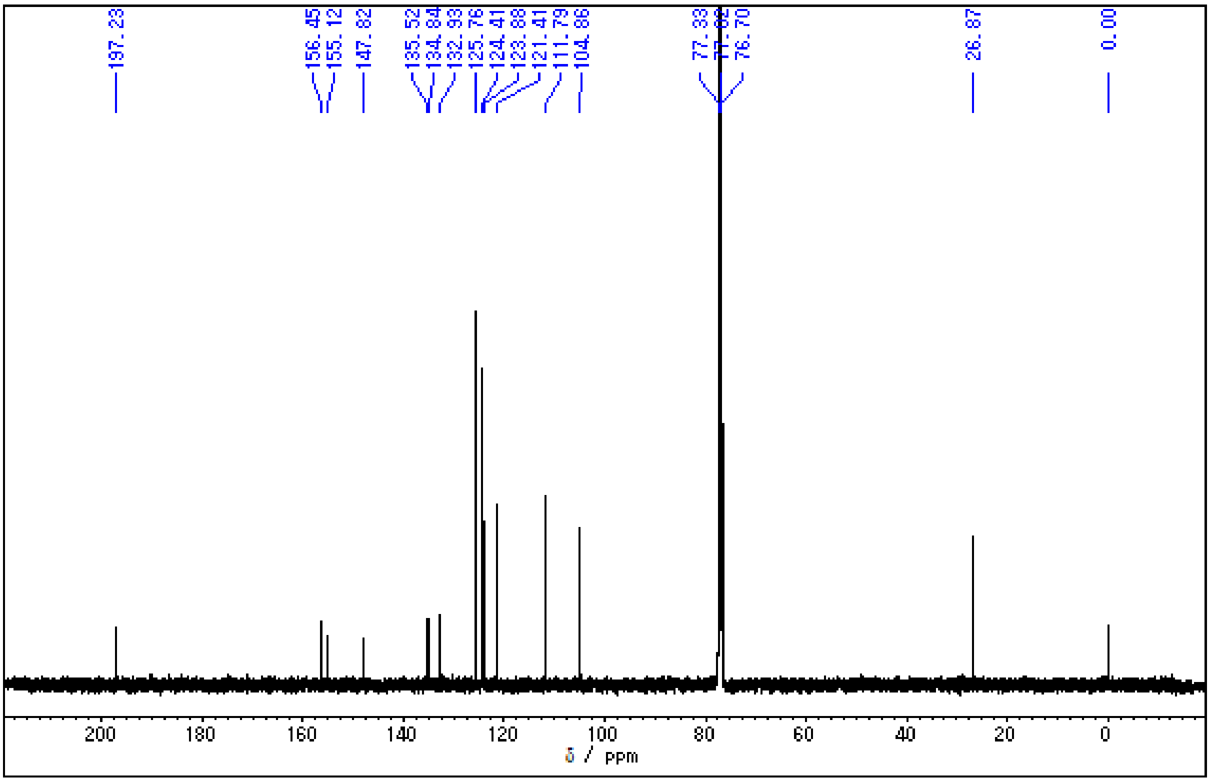

^{13}C NMR spectrum of **7a** (100 MHz, $CDCl_3$).

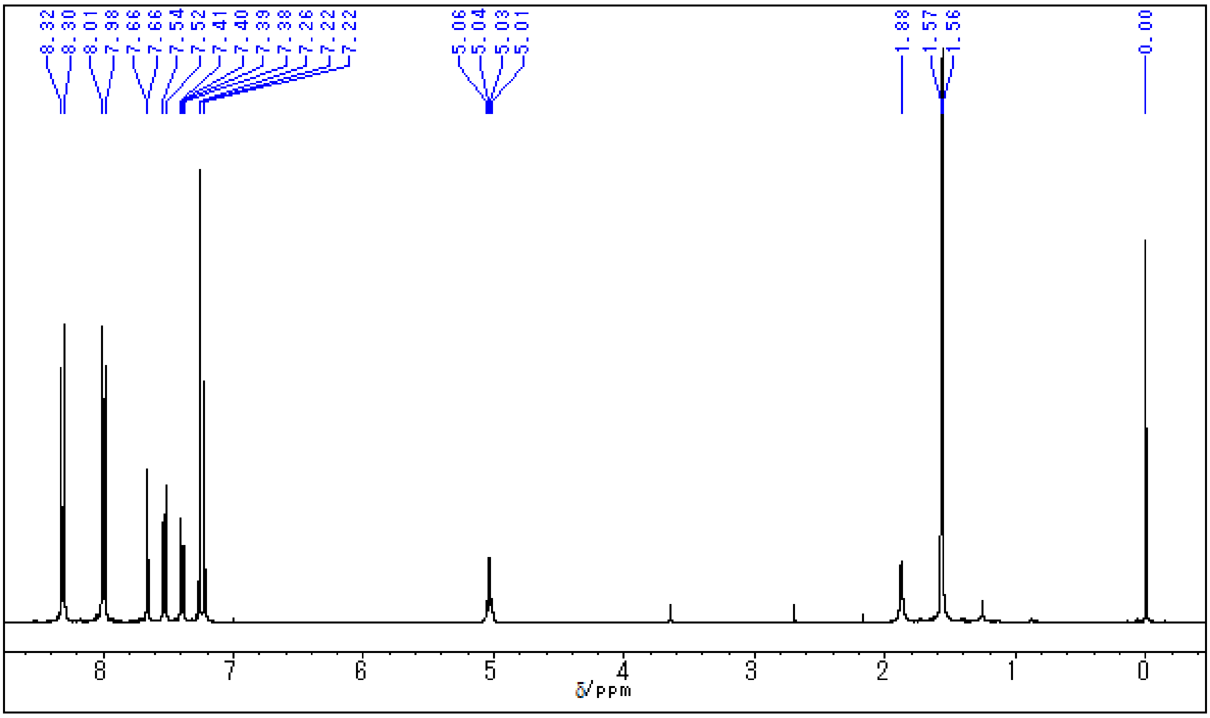

^{1}H NMR spectrum of **6b** (400 MHz, $CDCl_3$).

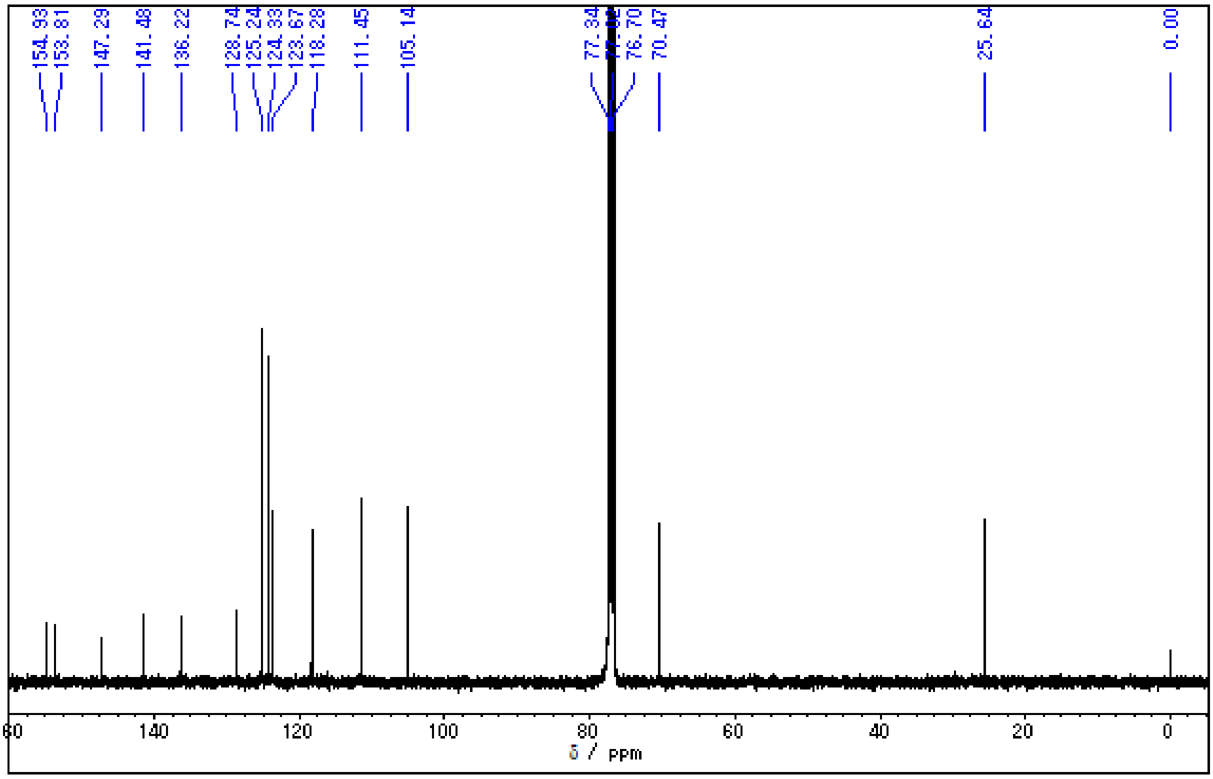

^{13}C NMR spectrum of **6b** (100 MHz, $CDCl_3$).

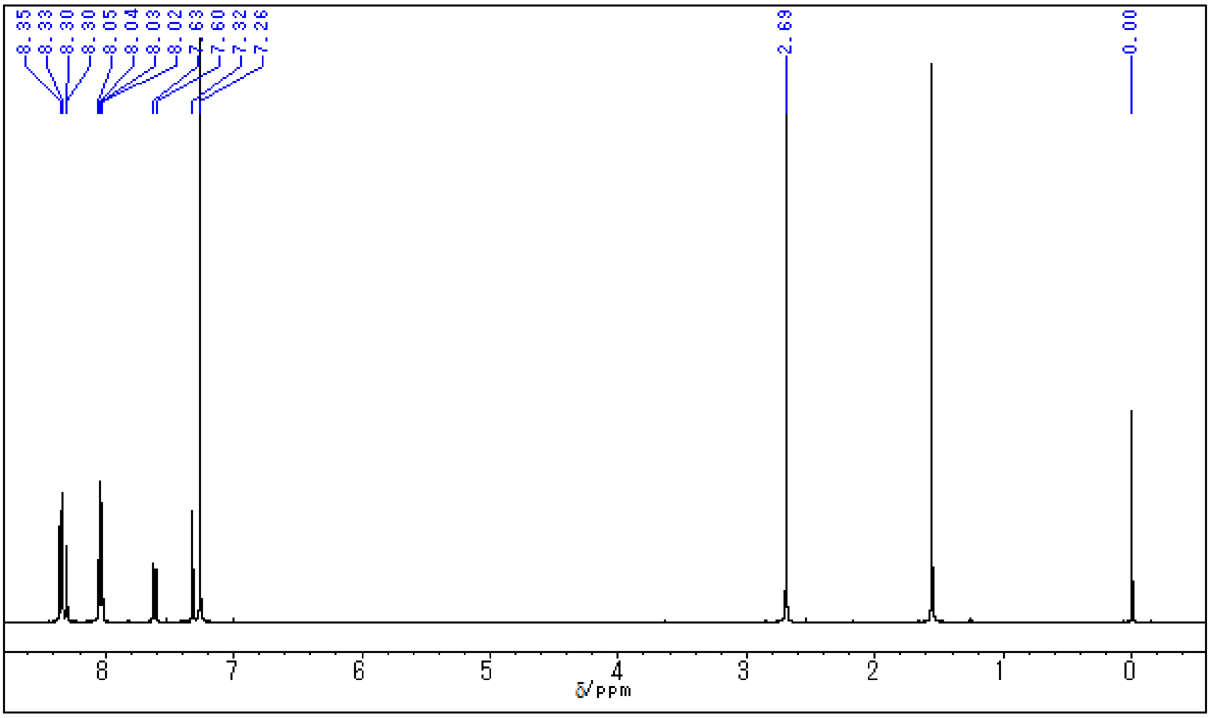

^{1}H NMR spectrum of **7b** (400 MHz, $CDCl_3$).

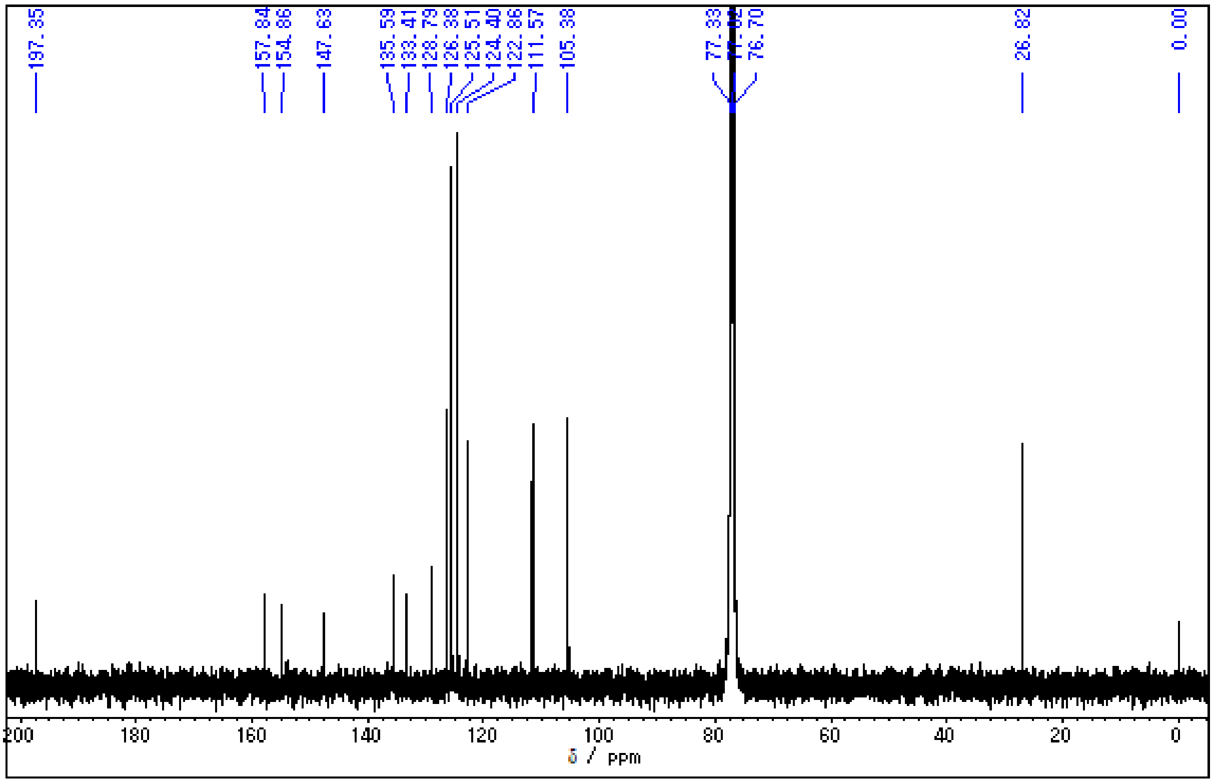

^{13}C NMR spectrum of **7b** (100 MHz, $CDCl_3$).

UV–vis absorption spectra of compounds

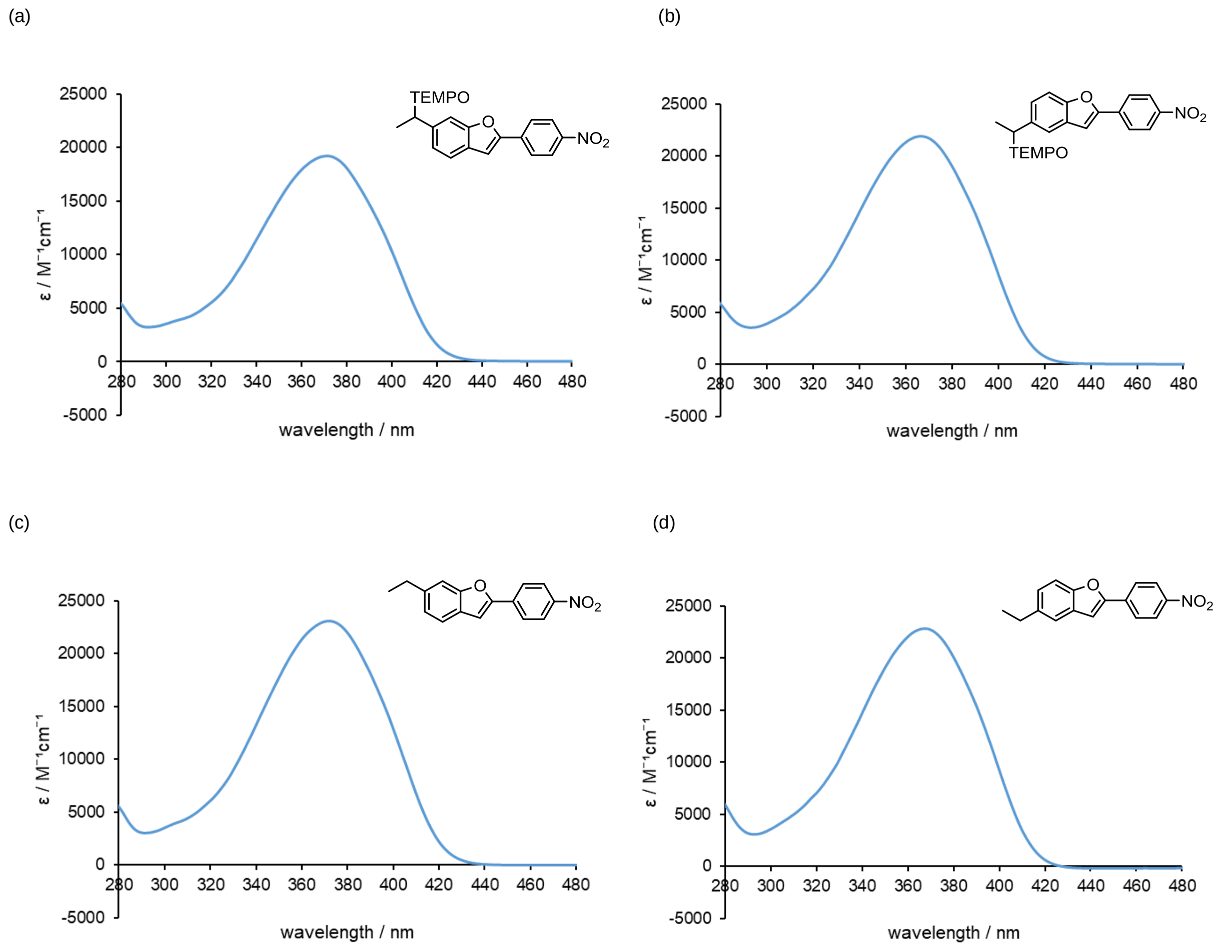

UV–vis absorption spectra in benzene of (a)**2a**, (b)**2b**, (c) **5a** and (d) **5b**.

The thermal stability of 2a and 2b

TEMPO was prepared to 1 mM, and compounds **2a** and **2b** prepared to 10 mM in benzene. 200 μl of the solution was poured in an ESR tube, and the temperature was changed from 290 to 340 K. The EPR measurement was carried out after every 10 minutes at each temperature.

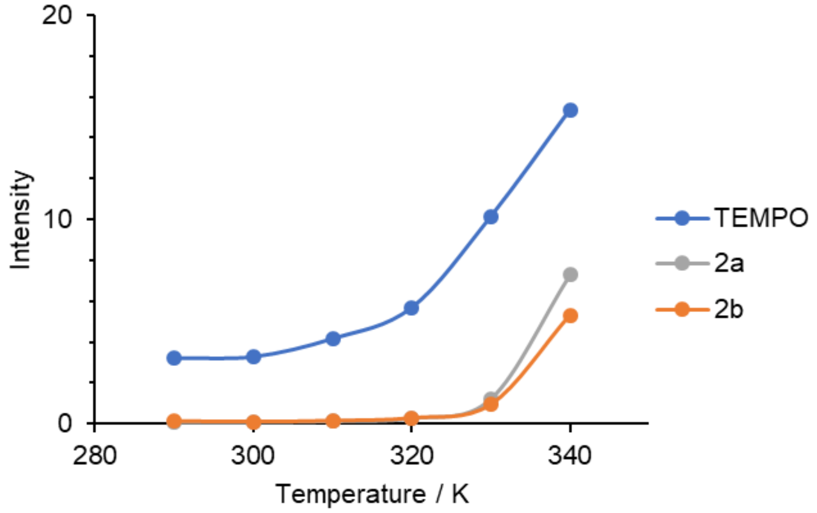

Figure S1. The thermal stability of **2a** and **2b** using ESR measurement.

The charge transfer transition of **5a**

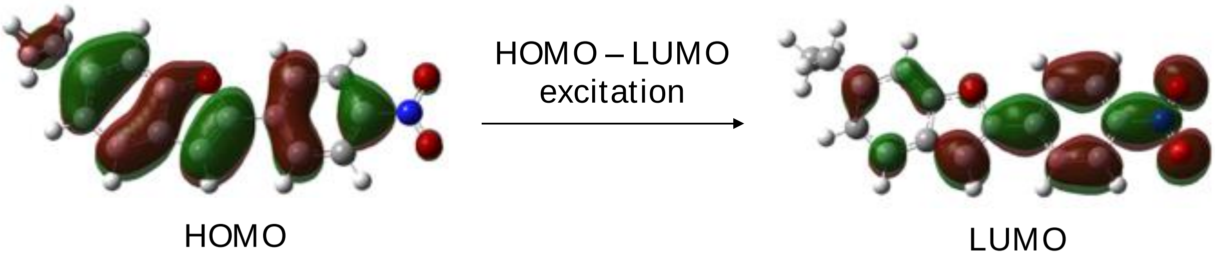

Figure S2. The charge transfer transition by the time-dependent density functional theory (TD-DFT) calculations of **5a** at the CAM-B3LYP/6-31G(d) level of theory.

The fluorescence lifetime of 2 and 5

Fluorescence decay measurements were performed using a time-correlated single-photon counting method. Laser excitation at 375 nm was performed using a diode laser (PicoQuant, LDH-P-C-375) with a power control unit (PicoQuant, PDL 800-B), with a repetition rate of 2.5 MHz. The temporal profiles of the fluorescence decays were detected by a microchannel plate photomultiplier (Hamamatsu, R3809U) equipped with a TCSPC computer board module (Becker and Hickl, SPC630). The full width at half maximum (fwhm) of the instrument response function was 51 ps. The values of χ^2 and the Durbin–Watson parameters were used to determine the quality of the fit obtained by nonlinear regression.

(a)

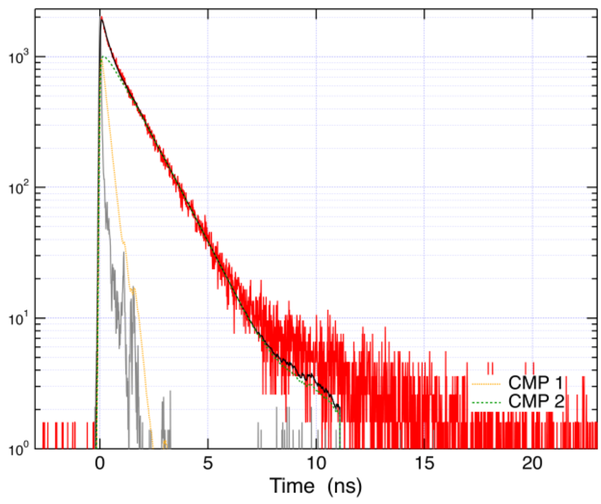

(b)

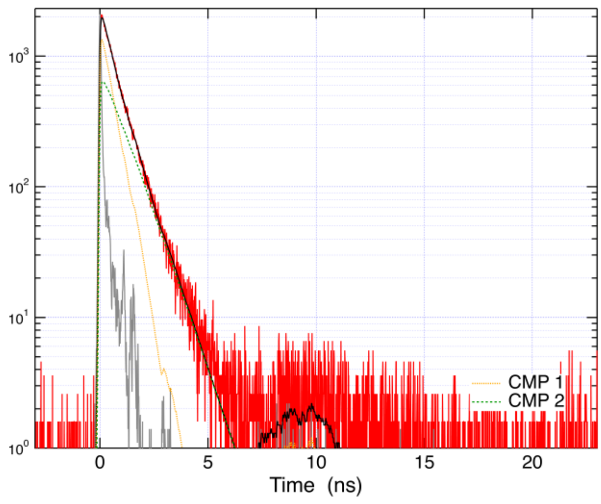

(c)

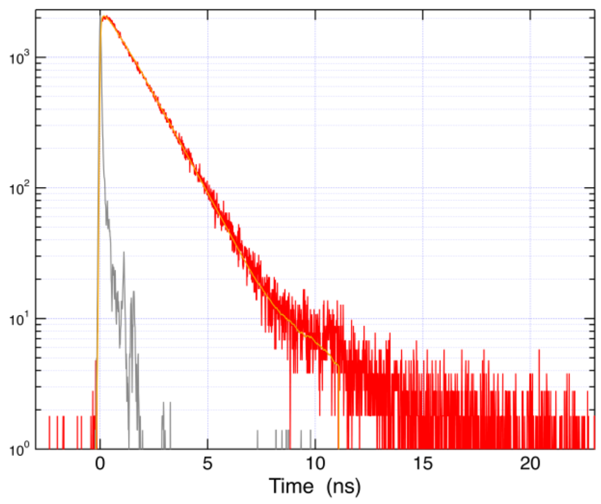

(d)

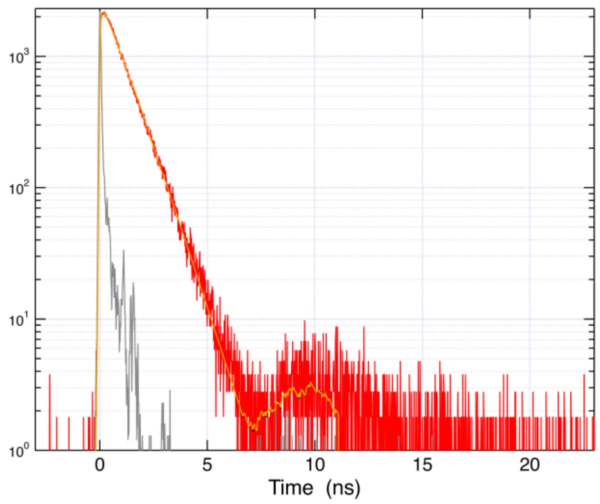

Figure S3. The fluorescence lifetime of (a) **2a**, (b) **2b**, (c) **5a** and (d) **5b** were determined by the exponential fitting.

Calibration curve of TEMPO

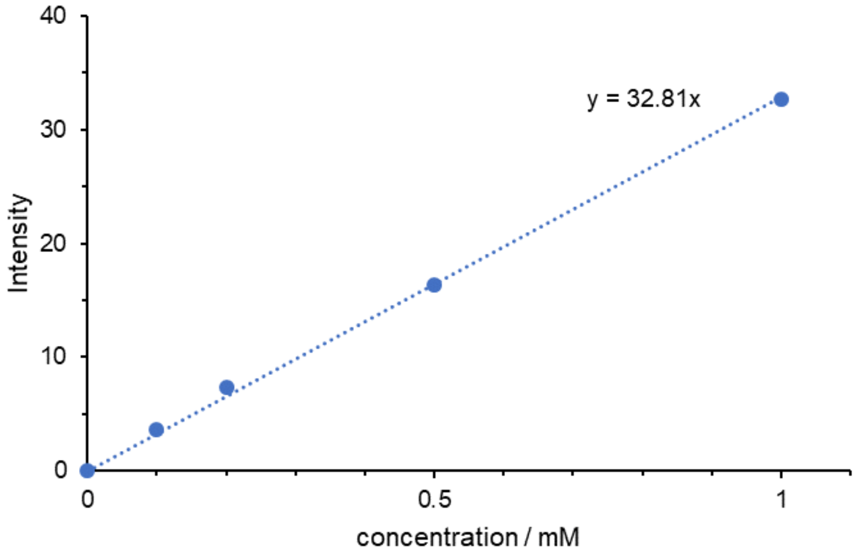

Figure S4. The calibration curve of the standard TEMPO by the EPR intensity

OP uncaging reaction of 2b

The compound **2b** was prepared to 5 mM in benzene. 200 μl of the solution was poured in an ESR tube, and the temperature was changed from 290 to 340 K. The EPR measurement was carried out after 1, 2.5, 5, 10 min irradiation by using xenon lamp (365 nm).

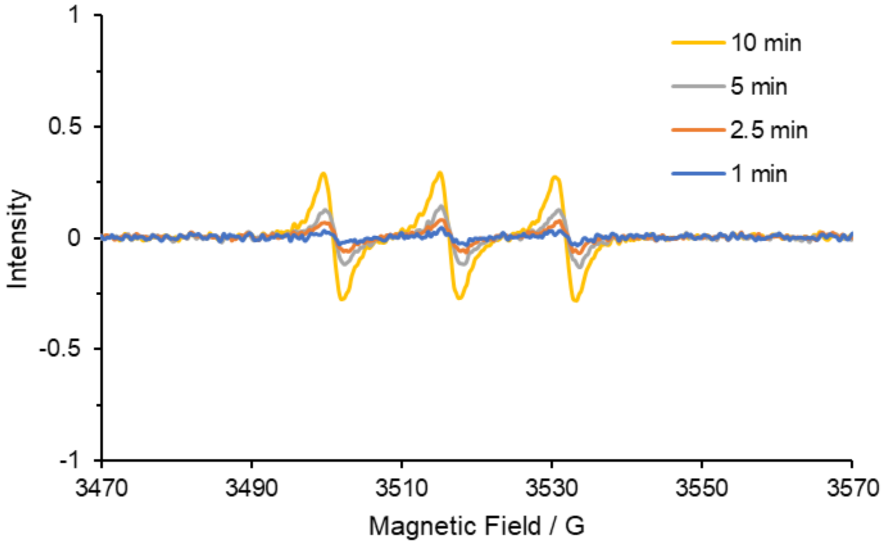

Figure S5. ESR spectra during the photolysis of **2b** (5 mM) in benzene using 365nm light.

TP uncaging reaction of 2b

TP uncaging reaction of **2b** was carried out in benzene using 710, 720, 730, 740, 750 and 760 nm light from a Ti:sapphire laser (pulse width 100 fs, 80 MHz) emitting at an average of 700 mW. The release of TEMPO upon photolysis was monitored by EPR measurment.

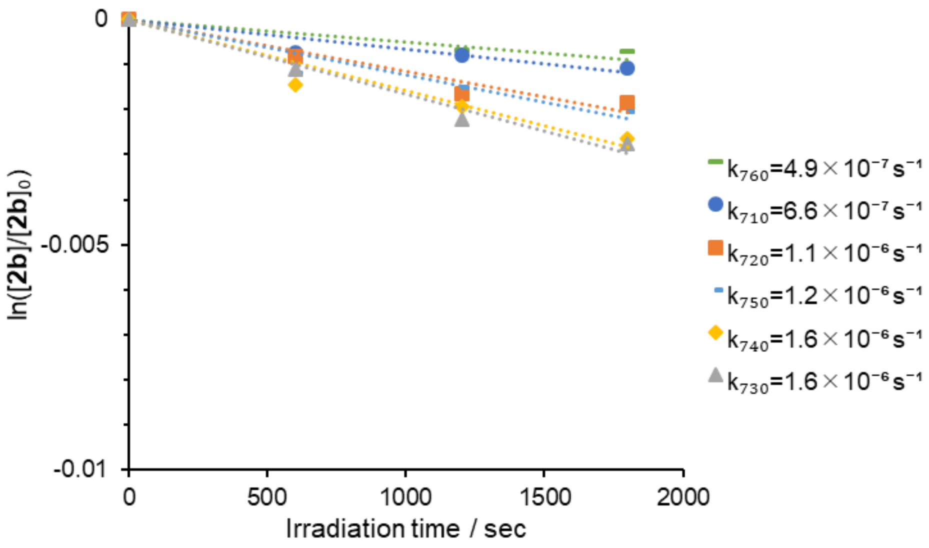

Figure S6. Time profile, ln([sub]/[sub]$_0$) versus irradiation time, of two-photon uncaging reaction of TEMPO in the photolysis of **2b** in benzene, at wavelength of 710–760 nm and at a power of 700 mW.

The typical EPR signals of TEMPO by the TP excitation

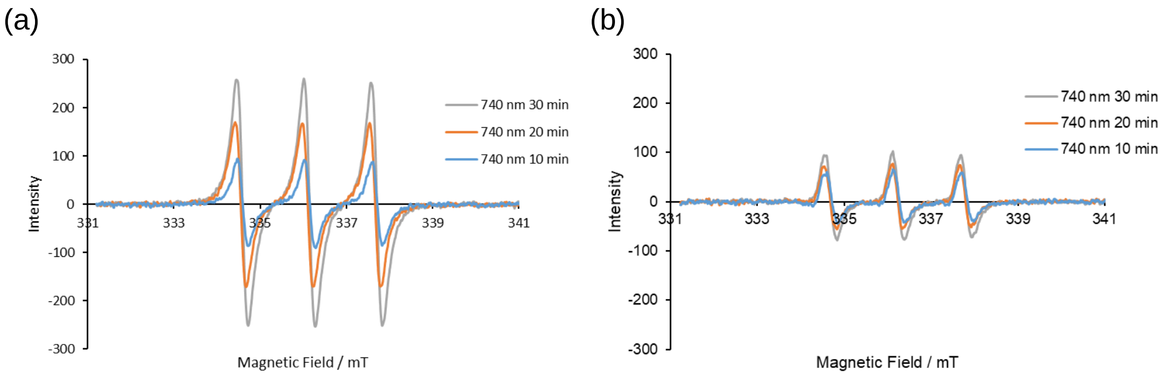

Figure S7. The typical EPR signals of TEMPO by the TP excitation of (a) **2a** and (b) **2b**.

Confirmation of cytotoxicity by caged radical compounds

One hundred thousand Lewis lung carcinoma (LLC) cells were seeded into 24-well plate (medium: DMEM) and incubated overnight at 37 °C in an atmosphere of 95% air and 5% CO_2. The medium was replaced with fresh phenol-red free DMEM containing various concentration of compound **2a** (0, 10, and 100 µg/mL) and further incubated for 4 hours under the same conditions without light exposure. Cell viability was determined by trypan blue exclusion. Bars represent the mean ± standard deviation (n = 4).

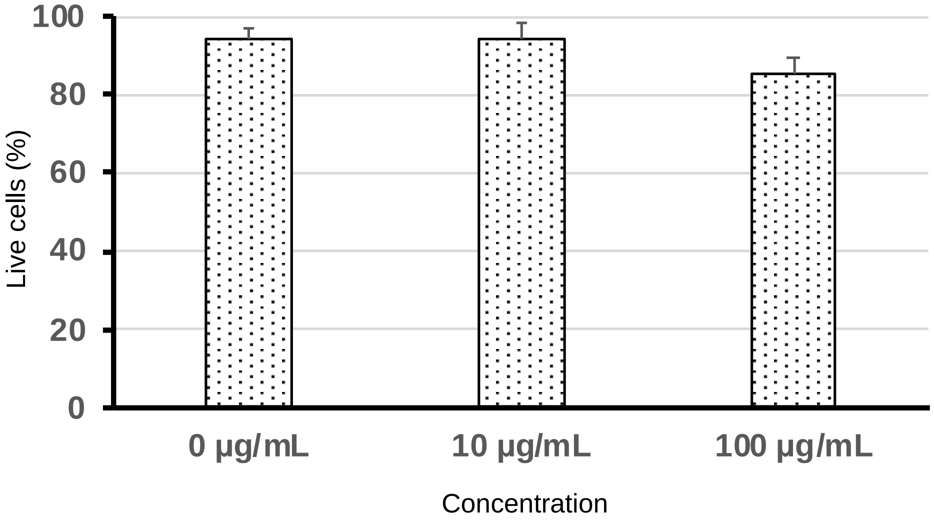

Figure S8. Cytotoxicity of **2a** itself without light exposure.

One hundred thousand Lewis lung carcinoma (LLC) cells were seeded into 24-well plate (medium: DMEM) and incubated overnight at 37 °C in an atmosphere of 95% air and 5% CO_2. The medium was replaced with fresh phenol-red free DMEM containing various concentration of compound **2a** (0, 1, 10, 30, 60, and 100 µg/mL). Four hours after 1 min exposure of 360 nm light using a fluorescence microscope (BIOREVO BZ-9000, Keyence, Osaka, Japan), cell viability was determined by trypan blue exclusion. Bars represent the mean ± standard deviation (n = 4).

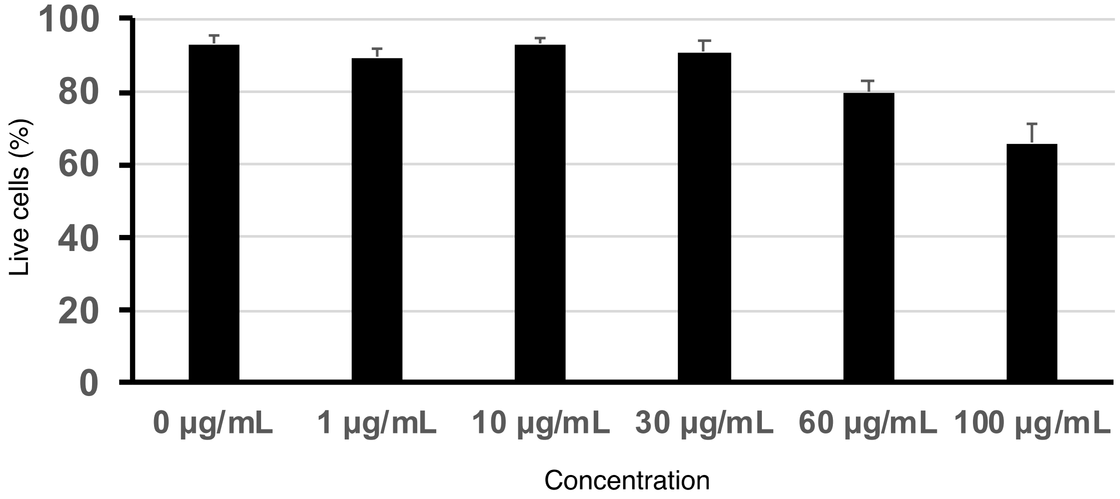

Figure S9. Dose-dependent cytocidal effect of **2a** with 360 nm light exposure.

One hundred thousand Lewis lung carcinoma (LLC) cells were seeded into 24-well plate (medium: DMEM) and incubated overnight at 37 °C in an atmosphere of 95% air and 5% CO_2. The medium was replaced with fresh phenol-red free DMEM containing 100 μg/mL of compound **2a**. Four hours after 1 min or no exposure of 360 nm light using a fluorescence microscope (BIOREVO BZ-9000, Keyence, Osaka, Japan), cell viability was determined by trypan blue exclusion. Bars represent the mean ± standard deviation (*n* = 4).

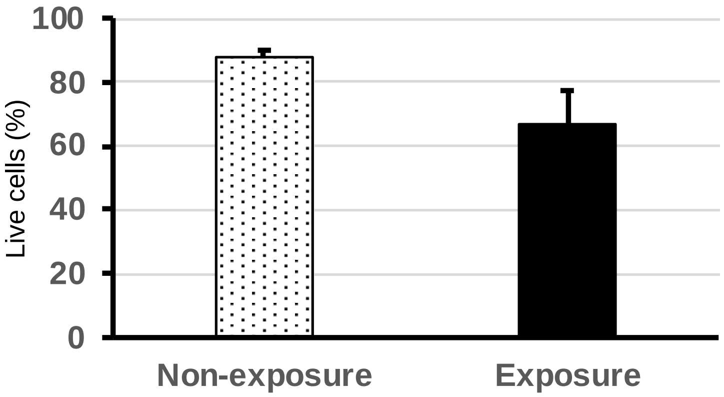

Figure S10. Photolysis-derived cytocidal effect of compound **2a**.
